# Prediction of *Sinorhizobium meliloti *sRNA genes and experimental detection in strain 2011

**DOI:** 10.1186/1471-2164-9-416

**Published:** 2008-09-16

**Authors:** Claudio Valverde, Jonathan Livny, Jan-Philip Schlüter, Jan Reinkensmeier, Anke Becker, Gustavo Parisi

**Affiliations:** 1 Programa Interacciones Biológicas, Departamento de Ciencia y Tecnología, Universidad Nacional de Quilmes, Roque Sáenz Peña 352, Bernal, Buenos Aires, B1876BXD, Argentina; 2 Channing Laboratories, Brigham and Women's Hospital, Harvard Medical School, 181 Longwood Avenue, Boston, MA 02115, USA; 3 Institute for Genome Research and Systems Biology, Center for Biotechnology (CeBiTec), Bielefeld University, 33594 Bielefeld, Germany; 4 Institute of Biology III, Faculty of Biology, University of Freiburg, Schänzlestr. 1, 79104 Freiburg, Germany; 5 Faculty of Technology, Bielefeld University, 33615 Bielefeld, Germany; 6 Grupo de Bioinformática Estructural, Centro de Estudios e Investigaciones, Universidad Nacional de Quilmes, Roque Saénz Peña 352, Bernal, Buenos Aires, B1876BXD, Argentina

## Abstract

**Background:**

Small non-coding RNAs (sRNAs) have emerged as ubiquitous regulatory elements in bacteria and other life domains. However, few sRNAs have been identified outside several well-studied species of gamma-proteobacteria and thus relatively little is known about the role of RNA-mediated regulation in most other bacterial genera. Here we have conducted a computational prediction of putative sRNA genes in intergenic regions (IgRs) of the symbiotic α-proteobacterium *S. meliloti *1021 and experimentally confirmed the expression of dozens of these candidate loci in the closely related strain *S. meliloti *2011.

**Results:**

Our first sRNA candidate compilation was based mainly on the output of the sRNAPredictHT algorithm. A thorough manual sequence analysis of the curated list rendered an initial set of 18 IgRs of interest, from which 14 candidates were detected in strain 2011 by Northern blot and/or microarray analysis. Interestingly, the intracellular transcript levels varied in response to various stress conditions. We developed an alternative computational method to more sensitively predict sRNA-encoding genes and score these predicted genes based on several features to allow identification of the strongest candidates. With this novel strategy, we predicted 60 chromosomal independent transcriptional units that, according to our annotation, represent strong candidates for sRNA-encoding genes, including most of the sRNAs experimentally verified in this work and in two other contemporary studies. Additionally, we predicted numerous candidate sRNA genes encoded in megaplasmids pSymA and pSymB. A significant proportion of the chromosomal- and megaplasmid-borne putative sRNA genes were validated by microarray analysis in strain 2011.

**Conclusion:**

Our data extend the number of experimentally detected *S. meliloti *sRNAs and significantly expand the list of putative sRNA-encoding IgRs in this and closely related α-proteobacteria. In addition, we have developed a computational method that proved useful to predict sRNA-encoding genes in *S. meliloti*. We anticipate that this predictive approach can be flexibly implemented in many other bacterial species.

## Background

In bacteria, small, non-coding RNA molecules that influence the expression of other genes are collectively referred to as sRNAs [1]. Significant experimental and theoretical evidence suggests sRNA-based regulation of gene expression is a paradigm common to all domains of life [2,3]. To date, two main mechanisms of sRNA activity have been described, both of which result in a modification of target mRNA translation and/or stability. The most common mechanism involves antisense pairing between the regulatory sRNA and the mRNA target [4]. In some cases, a single sRNA can mediate disparate regulatory effects on different mRNA targets. For instance, binding of the *E. coli *RyhB to the 5'-untranslated region of *shiA *mRNA activates *shiA *translation [5] whereas RhyB binding to *sodB *mRNA promotes its degradation [6]. In many cases the sRNA:mRNA interaction occurs over short regions of imperfect sequence complementarity and thus requires stabilization by the RNA chaperone Hfq [7]. The second sRNA-based mechanism is molecular mimicry, in which sRNAs offer multiple binding sites to RNA binding proteins of the CsrA/RsmA family, thus competitively relieving protein-mediated regulation of target mRNAs [8]. Most sRNAs characterized to date act as intermediate genetic elements of signal transduction cascades that are themselves initiated by a variety of external stimuli [9].

The number of putative and physically confirmed prokaryotic sRNAs has grown significantly in recent years, due in large part to the development and utilization of computational methods for predicting sRNA-encoding loci [10,11]. The pioneering predictive studies were initiated a few years ago when several groups discovered dozens of sRNAs in the intergenic regions of *E. coli *[12-14]. In these seminal studies, putative sRNAs were identified based on their association with genetic features common to several previously known sRNAs [15], such as their transcription from DNA regions between protein coding genes, their association with Rho-independent transcriptional terminator and/or promoter signals, the conservation of their primary sequence among closely related species, and their potential for encoding conserved secondary structure [16].

*Sinorhizobium meliloti *is an α-proteobacterium able to establish an intimate symbiosis with the roots of legumes belonging to the genera *Medicago*, *Melilotus *and *Trigonella *[17]. Upon an intricate chemical dialog and cross-recognition between bacterium and roots, *S. meliloti *colonizes the interior of *de novo *root organs, the nodules, in which it differentiates into bacteroids committed to biological fixation of atmospheric nitrogen [18]. The genome of the sequenced strain *S. meliloti *1021 is organized into three replicons, the "chromosome" (3.65 Mb) and two megaplasmids, pSymA (1.35 Mb) and pSymB (1.68 Mb), that were most likely acquired through horizontally transfer. Sequence analysis indicates that pSymA, the giant plasmid devoted to nodulation and nitrogen fixation functions, was acquired later in the evolution of the host bacterium than pSymB [19-21]. The chromosome of *S. meliloti *encodes an *hfq *homolog, suggesting that it also encodes sRNAs. However, prior to the initiation of this study, no screens for sRNAs had been conducted in *Sinorhizobium *and only the conserved chromosomal tmRNA homolog (*ssrA*) and an antisense countertranscript involved in control of pSymA and pSymB replication had been functionally characterized in *S. meliloti *[22-25]. While this work was in preparation, two groups reported the identification of a total of 15 chromosomally encoded sRNAs (including the widely conserved 6S RNA) and one pSymB-derived sRNA in *S. meliloti *strain 1021 [26,27]. These two studies employed similar predictive criteria, ones that were significantly different from the one utilized in this work. Here we report the prediction of dozens of putative sRNA genes encoded in the three replicons of *S. meliloti *and the experimental detection of many transcripts under different stress conditions in the closely related strain *S. meliloti *2011. Our first sRNA candidate compilation was based mainly on the output of the sRNAPredictHT algorithm. A thorough manual sequence analysis of the curated list rendered an initial set of 18 IgRs of interest, from which 14 candidates were detected by Northern blot and/or microarray analysis. As we suspected that *S. meliloti *would encode more sRNA transcripts, we developed an alternative computational method to more sensitively predict sRNA-encoding genes, which introduces a novel cumulative scoring procedure to identify the strongest candidates. This scheme takes into account the location of predicted transcription signatures (promoters and terminators), their relative orientations and proximity to flanking protein coding genes, and their association with regions of conserved primary sequence and secondary structure. A novel scoring algorithm was integrated into this approach to allow the strongest candidate loci to be readily identified. Using this prediction and scoring approach we detected most of the *S. meliloti *small transcripts revealed by our first screening and in two recent studies [26,27] as well as numerous strong candidates for novel sRNA-encoding genes in IgRs of *S. meliloti *chromosome and megaplasmids. A significant proportion of these chromosomal- and megaplasmid-borne putative sRNA genes were validated by microarray analysis.

## Methods

### First set of predicted sRNA-encoding genes in *S. meliloti *chromosomal intergenic regions

Among the 2920 chromosomal IgRs of *S. meliloti *1021 [28], a first set of IgRs potentially encoding sRNAs (Table 1) was compiled by: 1) selection of IgRs with annotated orphan transcriptional terminators [28]; 2) selection of IgRs in the vicinity of tRNAs [29,30]; 3) application of sRNAPredictHT (J. Livny; unpublished data), an improved version of the program sRNApredict2 developed by Livny and co-workers [31]. Using default parameters, sRNAPredictHT identified 186 sequence elements as putative sRNAs (Additional file 1). However, almost 60% of the hits corresponded to annotated [19,28,32] or non-annotated sequence repeats. Each IgR was used to query Rfam database [33] to identify previously annotated RNA regulatory elements and then inspected for the presence of transcriptional signals (promoters and Rho-independent terminators; see below). We retained 17 chromosomal IgRs that were likely to encode sRNAs and an additional IgR encoding a putative 6S RNA homologue (Table 1).

**Table 1:** First compilation of *S. meliloti *chromosomal IgRs predicted to encode sRNAs.

**IgR#**	**Found ** **by^1^**	**Adjacent ** **genes^2^**	**sRNA ** **strand^2^**	**Prom.?^3^**	**Term.?^3^**	**Blast hits ** **in^4^**	**Expected ** **size^5^**	**Band size in ** **Northern blot**	**Microarray ** **detection^7^**	**Gene^8^**	**Reference**
1	Rfam	*SMc03975> SMc03976<*	<	s70	n	eubacteria	~160 nt	~155 nt	<, H, C, S, A, B, O (2972187–2972236)	6S RNA (*smrC22 *= *sra56*)	[26,27,62,67]

2	OT	*SMc04042< SMc04043<*	>	NN – s70	y	*Sm Rl Re At*	~80 nt	duplet < 100 nt	-	*sm8 *= *suhB*	[65,67], this work

3	SP	*SMc01933< proS>*	<	NN – s70	y	*Sm Re Rl At Ml*	~150 nt	~165 nt	<, A, C, H, S (1398340–1398389 1398389–1398340)	*smrC9 *= *sra32*	[26,27]

4	tR	*Gln-tRNA< SMc00810>*	<	-	y	*Sm Re Rl*	?	multiple ~150 $~& 80 nt mainly	-	*sm137*	this work

5	tR	*SMc02151< Thr-tRNA<*	>	NN – s70	y	-	~240 nt	no bands detected	>, S (*) (560802–560753)	*sra12*	[27]

6	OT	*gltB< SMc04029>*	>	NN	y	*Sm Re Rl*	~100–200 nt	not done ^6^	>, A, C (3031662–3031711)	-	this work

7	OT	*SMc04453< SMc01885>*	<	NN	y	*Sm Re Rl*	~390 nt	duplet, ~135 & ~200 nt	<, A, C, O, S (2321429–2321478)	*sm26*	this work

8	OT	*SMc01257< sda>*	>	NN – s70 – PhoB	y	*Sm At Oa Msp*	~170 nt	not done ^6^	-	*sm30*	this work

9	OT	*thdF< rho<*	>	s70	y	*Sm*	~180 nt	not done	-	*sm130*	this work

10	SP	*SMc00034> SMc00096<*	>	s70	y	*Sm Re Rl*	~190 nt	~175 nt	-	*sra25*	[27]

11	SP	*SMc00108< SMc00109<*	<	s70	y	*Sm Rl Re At*	~100–120 nt	duplet, ~120 $~& 110 nt	-	*sm145*	this work

12	SP	*celR2> rpmG<*	>	s70	y	*Sm Rl Re At Ml Msp Ac*	~150 nt	not done ^6^	>, A, B, C, H, O, S (1411684–1411733 1411738–1411689)	*smrC10 *= *sra33*	[26,27]

13	SP	*polA< SMc02851>*	>	NN – s70	y	*Sm Rl Re At*	~150 nt	duplet, ~140 & ~90 nt	-	*smrC7 *= *sra03*	[26,27]

14	SP	*SMc02910< SMc02911>*	>	s70	y	*Sm Re Rl At Ml Bo Oa*	~60–150 nt	multiple, ~90–100 & ~180–210	>, B, C, O, (267010–267059 267059–267010)	*sm76*	this work

15	SP	*SMc03988> SMc03989>*	>	s70	y	*Sm Rl Re At*	~110 nt	~80 nt	>, A, B, C, H, O, S (2986452–2986501 2986508–2986459)	*sm84*	this work

16	SP	*atpH< SMc02497<*	>	s70	y	*Sm Rl Re At*	~110 nt	~100 nt	-	*sm270*	this work

17	SP	*dapF< ffh>*	>	s70	y	*Sm Rl Re At*	~120 nt	not done	>, A, B, O, S (3522279–3522328)	*sm5*	this work

18	OT	*SMc00821> SMc00822>*	>	-	y	*Sm Re Rl*	~80 nt	not done	>, A, C, H, O, S (843459–843508 843508–843459)	*sm190*	this work

^1 ^Rfam, RNA families database [33,67]; OT, IgR with annotated orphan terminator; tR, IgR flanking tRNAs; SP, sRNAPredictHT.

^2 ^The transcribed strand is indicated (>, forward; <, reverse)

^3 ^Putative promoter sequence detected by conservation in sequence alignment, search of consensus sequences or neural network promoter prediction. s70, sequence highly similar to the *S. meliloti *constitutive promoter consensus sequence [49]; NN, neural network promoter prediction [47]; PhoB, sequence detected with homology to the PhoB transcriptional regulator binding consensus [51].

^4 ^Sm, *Sinorhizobium medicae *WSM419; Rl, *Rhizobium leguminosarum *biovar *viciae *3841; Re, *Rhizobium etli *CFN42; At, *Agrobacterium tumefaciens *C58; Ml, *Mesorhizobium loti *MAFF303099; Oa, *Ochrobactrum anthropi *ATCC49188; Msp, *Mesorhizobium *sp. BNC1; Bo, *Brucella ovis *ATCC25840.

^5 ^Approximate length of sRNA based on putative 5' and 3' ends.

^6 ^PCR failed to amplify the IgR.

^7 ^Detection on the oligonucleotide microarray Sm14kOLI. > and < denote the orientation of the detected signal (absolute M-value ≥ 2.5 represents an enrichment of small RNA transcripts). A, B, C, H, O and S denote the stress condition under which the signal was detected (A, acidic; B, basic; C, cold shock; H, heat shock; O, oxidative, S, saline). The coordinates of the oligonucleotide probes that gave positive signals are indicated in brackets. -, not detected under the conditions studied. (*), M-value ≥ 2.

^8 ^Gene name as designated in the literature (*smrC*#, *sra*#) [26,27]or according to our predictive scheme (*sm#*).

### Northern blot detection of the first set of sRNA candidates

*Sinorhizobium meliloti *strain 2011 [34] was maintained on TY agar plates [35] with streptomycin (400 μg/ml). We chose the *Rhizobium *defined medium (RDM) [36] with shaking (120 rpm) at 28°C as the referential growth condition. For preparation of RNA extracts, 125-ml flasks containing 20 ml of RDM were inoculated with 0.2 ml of a saturated RDM pre-culture and incubated at 120 rpm until cell harvest. To introduce stress conditions, the RDM basal medium or growth conditions were modified as follows: high salt RDM (0.3 M NaCl), low phosphate RDM (0.1 mM phosphate, 10 mM MOPS pH 7.0), RDM with ethanol (2% v/v), RDM with SDS (0.1% w/v) and RDM with H_2_O_2 _(0.1 mM). High temperature stress was applied by growing cells at 37°C. For acid stress, exponential phase cells growing in 20 ml of RDM (OD_600 _= 0.5) were collected by low speed centrifugation, washed with and resuspended in 20 ml of RDM containing 20 mM MES and equilibrated at pH 5.5, and incubated 90 min at 28°C with shaking before harvesting cells for RNA extraction.

Total RNA was extracted immediately after cell harvest by low speed centrifugation (1800 g, 10 min, and 20°C). The cell pellet was resuspended in Trizol^® ^(Invitrogen; 1.5 ml for cultures with OD_600 _< 1.5 or 3.0 ml for cultures with OD_600 _> 1.5) and treated 1 min at 60°C. Upon addition of 0.2 vols of chloroform and vigorous shaking during 15 secs, the RNA present in the aqueous supernatant was precipitated with 0.5 vol of isopropanol. The pellet was washed in 70% ethanol, air dried and resuspended in 20 μl of DEPC-treated deionized water. RNA samples were conserved at -130°C. The purity and integrity of RNA preparations were assessed by denaturing PAGE electrophoresis followed by silver staining [37] and the RNA concentration was estimated by UV spectrometry [38]. For Northern blots, 1–3 μg RNA present in 1 μl of each sample were fractionated on denaturing polyacrylamide gels (60 × 80 × 0.75 mm containing 8.3 M urea, 8% acrylamide and 0.2% bisacrylamide in 1× TBE buffer). The lane corresponding to the molecular weight markers (low range RNA ladder; Fermentas) was cut out, stained with 5 μg ml^-1 ^ethidium bromide and photographed under UV light. The rest of the gel was electroblotted at 150 mA (15–25 V) onto a Hybond-N membrane in 1× TBE buffer for 20 min. Membranes were washed with 2× SSC (0.3 M NaCl and 30 mM sodium citrate) before nucleic acids were cross-linked by exposure to UV light for 5 min [38]. Northern hybridizations were done with digoxigenin (DIG)-labeled DNA probes generated by PCR covering entirely or partially each IgR (Additional file 2). The IgR amplicons of detected candidate sRNAs were cloned in the pCR^®^2.1-TOPO vector and sequenced to confirm the identity of the PCR products. Hybridized membranes were developed following the protocol recommended by the manufacturer (Roche Diagnostics GmbH). The detected RNA bands were quantified by densitometry with ImageJ v1.38 [39] and standardized by the amount of loaded RNA visualized by silver staining.

### Microarray detection of sRNA candidates

Pre-cultures of *S. meliloti *strain 2011 were grown at 30°C in TY [35] or GMS [40] media. For RNA isolation, 100 ml flasks with 50 ml TY or GMS medium, supplemented with 8 μg/ml nalidixic acid, were inoculated with 200 μl of pre-culture and incubated in a rotary shaker (175 rpm) at 30°C to an OD_600 _= 0.6. To induce stress, the medium and growth conditions were modified as follows. High salt stress: addition of NaCl to a final concentration of 0.4 M in GMS medium. Oxidative stress: addition of H_2_O_2 _to a final concentration of 10 mM in GMS medium. Cold shock stress: temperature shift of the culture in TY medium from 30°C to 20°C. Heat shock stress: temperature shift of the culture in TY medium from 30°C to 40°C. Acid or alkaline stress: cultures grown in GMS to an OD_600 _= 0.6 were centrifuged and then re-suspended in GMS modified by adding HCl to pH 5.8, or by adding NaOH to pH 8.5. In all cases, cells were harvested 15 and 45 min after exposure to stress conditions.

RNA was isolated and separated into small RNA (< 200 nt) and long RNA (> 200 nt) fractions using the miRNeasy Mini Kit (Qiagen) or Ambion mirVana miRNA Isolation Kit (Ambion) according to the manufacturers' instructions. Quality of RNA was analyzed applying the Agilent RNA 6000 Pico Kit on the Agilent 2100 Bioanalyzer (Agilent Technologies). To consider both orientations, aliquots from the same fractions of small and long RNA pools were sense labelled using the mirVana miRNA Labeling Kit (Ambion) and antisense labelled as described [41]. Differing from the cDNA labelling procedure [41], small RNA fractions were first tailed with PolyA polymerase (Ambion). Oligo dT and amino-allyl random hexamer primers were used for the synthesis of cDNA.

Hybridization of the small RNA fraction (Cy3-fluorescent marker) was compared to that of the long RNA fraction (Cy5-fluorescent marker). Three combinations were performed: 1. the small RNA fraction with the long RNA fraction, both of which were sense labelled, 2. the same fractions in which both were antisense labelled, and 3. a combination of the sense labelled small RNA fraction and the antisense labelled long RNA fraction. Slide processing, sample hybridization, and scanning procedures were performed as described [41] applying the Sm14kOLI microarray that carries 50 mer to 70 mer oligonucleotide probed directed against coding regions and intergenic regions [42]. Analysis of microarray images was carried out applying the ImaGene 6.0 software (BioDiscoveries) [41]. Lowess normalization and significance test (fdr) were performed with the EMMA software [43]. The M-Value represents the logarithmic ratio between both channels. The A-Value represents the logarithm of the combined intensities of both channels. Positive M-values ≥ 2.5 represent an enrichment of small RNA fragments (< = 200 nt) and therefore were classified as sRNA candidates.

### Novel method for in silico identification of sRNA candidate genes

From the original 2920 chromosomal IgRs, all the annotated repetitive elements of 1021 chromosome (Sm-repeats, RIMEs and AB, C motifs) [19,28,32] were removed and the flanking IgR segments were treated as new IgRs. 1720 chromosomal IgRs free of annotated repeats and longer than 150 nt were retained for further analysis. Certain IgRs were also removed if they gave BlastN hits with E-value < 10^-3 ^when queried against themselves, reducing the number of IgRs to 778. With the help of open source algorithms and web based tools, the 778 chromosomal IgRs were subjected to the following sequence analyses: prediction of Rho-independent transcription terminators and of promoter signals, sequence conservation (BlastN; [44]) and secondary structure conservation (QRNA analysis) [45].

For prediction of Rho-independent transcription terminators, the web based TranstermHP server [46] was queried to generate a list of putative terminator sequences in chromosomal IgRs of strain 1021, having a stem length of 4–23 bases, a hairpin score ≤ -1.5, a tail score ≤ -2.0 and ≥ 80% of confidence. Orphan terminators (i.e., those that do not correspond to flanking CDS) were scored 3. Predicted terminators co-oriented with flanking ORFs were scored according to their relative distance to the 3'-end of the corresponding annotated gene so that a score of 2 was assigned if the terminator was farther than 200 bp, 1 if the distance was 100–200 nt, and 0 if it was closer than 100 bp.

Promoter signals were predicted with three alternative methods. A first set of putative promoters was generated with a web based neural network based routine [47] set up for bacterial sequences in both DNA strands with a minimum score of 0.8. A second set of putative promoters was compiled by querying IgRs with Fuzznuc [48] using available *S. meliloti *consensus sequences as input. For σ^70^-dependent promoters the query was CTTGAC(N_17_)CTATAT [49] with up to 4 mismatches allowed. For σ^54^-dependent promoters the query was TGGCACG(N_4_)TTGCW [50] with up to 2 mismatches allowed. For putative PhoB-binding sites the results of two queries were pooled, CTGTCAT(N_4_)CTGTCAT [51] with up to 4 mismatches allowed and TGWCAM(N_4_)CYKTCAK [52] with up to 2 mismatches allowed. A third group of promoters was predicted with the help of the matrix-scan tool available at the Rsat web server [53], upon introduction of available scoring matrices for *S. meliloti *σ^70^-, σ^54^- and PhoB-dependent promoters [49-52] and with default parameters. A similar scoring criterion to that used for terminators was applied to predicted promoters. Orphan promoters were scored 3. Putative promoters were rated 2 if the 5'-end of the co-oriented flanking CDS was farther than 300 bp, 1 if this distance was 200–300 bp and 0, if they were closer than 200 bp.

Similarity searches performed with BlastN were done using default parameter values. IgRs were used to query against a database of 559 complete eubacterial genomes [54] and we defined a Blast score (#BlastN) that for each input IgR sequence consists in the sum of all the hits with E-values below 10^-3^. We used QRNA [45] to analyze the sequence alignments generated for each IgR and a score was derived summing all the positive hits detected (#QRNA).

Finally, the individual scores for predicted terminators (#T), promoters (#P), BlastN (#BlastN) and QRNA analysis (#QRNA) were combined to generate a Global Score (GS). If a putative promoter and a terminator lay co-oriented and separated from each other by 40–500 bp, suggesting the presence of a single and independent transcriptional unit, the IgR is scored 10 and the individual scores for promoter and terminator are no longer considered. The GS for those IgRs containing such putative elements indicative of sRNAs was calculated as (10 + #BlastN + #QRNA). For those IgRs lacking putative independent transcriptional units, the GS was calculated as (#T + #P + #BlastN + #QRNA).

## Results & Discussion

### A first selection of chromosomal intergenic regions potentially encoding sRNAs

At the time we initiated this study, the only chromosomal non-coding RNA gene that had been characterized in the α-proteobacterium *S. meliloti *was the tmRNA homolog *ssrA *[23]. However, several findings suggested that other sRNAs might be expressed in this α-proteobacterium. The electrophoretic fractionation in denaturing polyacrylamide gels of total RNA from strain 2011 cells grown under different conditions (Additional file 3) revealed several RNA bands of < 300 nt other than the conserved and abundant 5S RNA, 4.5S RNA and tRNAs [27]. Another indirect evidence of the existence of sRNAs in *S. meliloti *comes from the pleiotropic phenotype of the *S. meliloti *2011*hfq *mutant (Sobrero & Valverde, unpublished). These observations suggest that the product of the *hfq *gene (SMc01048 = *nrfA*) may be required to assist diverse regulatory interactions between mRNAs and sRNAs, as reported for other bacterial species [7,55]. We thus decided to perform a bioinformatic search of sRNA genes using the genomic information of the sequenced strain *S. meliloti *1021.

Although there are reports of sRNAs transcribed from coding regions in other bacteria [56,57], we focused our search in the regions between annotated ORFs (hereafter IgRs) of the *S. meliloti *chromosome [19]. We first identified in the *S. meliloti *annotated database [28] chromosomal IgRs containing transcriptional terminators unlikely to be associated with flanking ORFs as well as regions of sequence conservation in the vicinity of annotated tRNA genes, which may represent horizontally transferred genetic elements [29,30]. This "manual" procedure resulted in the identification of a few interesting IgRs (tagged OT and tR in Table 1). Next, we applied sRNAPredictHT, an improved version of the systematic and integrative tool sRNApredict2 already used for the prediction of sRNA genes in several bacterial species [31]. sRNAPredictHT identifies sRNA-encoding loci based on the co-localization of transcriptional terminators and IgR sequence conservation [31]. Among the 186 candidate loci identified by sRNAPredictHT (Additional file 1), 56% were identified in IgRs containing at least one repetitive DNA element, either the annotated *Rhizobium*-specific intergenic mosaic elements (RIMEs) [19,32], Sm-repeats [19,28], AB, C palindromes [19,28], or in some cases, even non-annotated repeats. Rhizobial genomes are characterized for the presence of dozens of these intergenic sequences of unknown function that typically share significant primary sequence and secondary structure conservation [58]. Upon elimination of IgRs containing repeats, the sRNAPredictHT output was narrowed down to a list of 76 candidate IgRs (Additional file 4). To further reduce the number of IgRs for experimental verification, we looked for candidates associated with putative promoters. This stringent filtering yielded a list of 17 interesting IgRs (Table 1). In fact, 15 candidate IgRs have both potential 5' and 3' transcriptional signals and are conserved in related species (Table 1), suggesting that they correspond to *bona fide *sRNA-encoding genes. Table 1 also includes a putative homolog of the widely conserved 6S RNA (IgR#1; [33]) which was not picked up by sRNAPredictHT because it lacks a typical Rho-independent terminator (Table 1). With the exception of IgR#5, all the candidates in Table 1 are conserved in at least one related α-proteobacterium. All IgRs but the aforementioned IgR#1 (6S RNA) are associated with a predicted Rho-independent terminator.

### Experimental verification of selected sRNA candidates in *S. meliloti *strain 2011

For experimental verification of most putative sRNA genes listed in Table 1, we performed Northern hybridizations and microarray analysis of RNA from *S. meliloti *strain 2011 that, like the sequenced strain 1021, is a streptomycin-resistant mutant derived from the isolate SU47. Although the separate and parallel continuous manipulation of these isogenic strains gave origin to subtle differences in their symbiotic behaviour and gene expression [52,59], the overall high degree of sequence similarity between both strains permits the use of strain 2011 to test predictions based on 1021 sequence. As many characterized sRNAs are involved in regulatory processes induced by a variety of external stimuli [9], RNA extracts were prepared from cells grown both under standard culture conditions and under a variety of stressful conditions.

**Figure 1:**
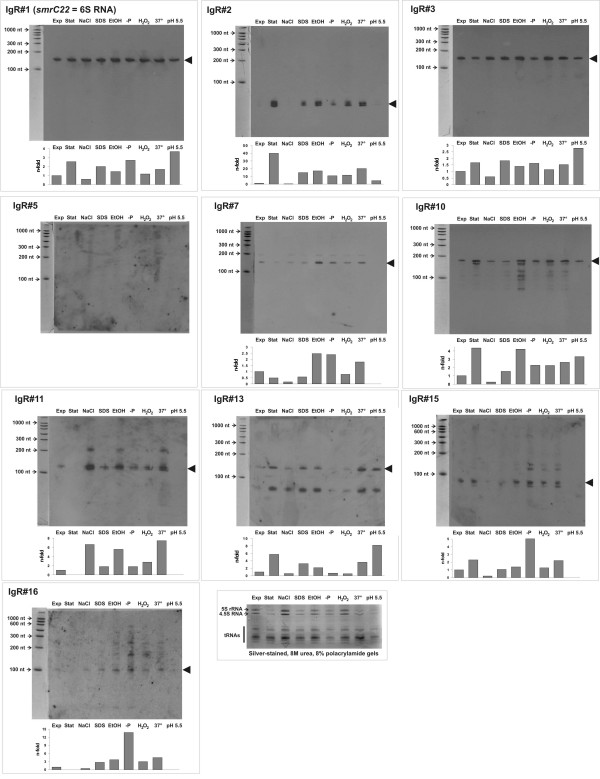
**Northern blot analysis of putative sRNAs encoded in the chromosome of *S. meliloti *strain 2011**. Total RNA was isolated from *S. meliloti *2011 cells grown at 28°C with agitation (120 rpm) in RDM minimal medium and harvested at OD_600 _= 0.5 (Exp) or at OD_600 _= 3.9 (Stat). Total RNA was also isolated from cells subjected to high salt stress (NaCl; 0.3 M NaCl in RDM, OD_600 _= 0.55), membrane stress (EtOH; RDM with 2% v/v ethanol, OD_600 _= 1.6; or SDS; RDM with 0.1% w/v SDS; OD_600 _= 1.0), phosphate starvation (-P; RDM with 0.1 mM phosphate and 10 mM MOPS pH 7.0, OD_600 _= 1.0), oxidative stress (H_2_O_2_; RDM with 0.1 mM H_2_O_2_, OD_600 _= 1.1), heat stress (37°; RDM grown at 37°C, OD_600 _= 0.95) and acid stress (pH 5.5; treatment of exponential phase cells at OD_600 _= 0.5 during 90 min at pH 5.5 before harvest). Northern hybridizations were done with PCR-generated digoxigenin-labeled dsDNA probes directed against the entire IgR or an internal fragment (see Figure 2 and Additional file 2 for further details). RNA molecular weight markers (with their sizes indicated in nt with small arrows at the left of each panel) were run with each set of RNA samples for estimation of transcript size. As exposure times were optimized for visualization here, the signal intensity does not indicate relative abundance of detected transcripts between different IgRs. Hybridization signals were quantified with ImageJ software, normalized to the amount of 5S RNA, 4.5S RNA and tRNA bands detected in silver stained gels present in each sample (bottom panel) and plotted in a bar graph shown below each Northern blot. The band intensity units are relative to the normalized amount present in Exp cells, which were set as 1.0.

Of the 12 candidate IgRs from our initial compilation that were subjected to experimental verification by Northern analysis of *S. meliloti *2011 RNA, 11 were detected (Table 1, Figure 1, Additional file 5). For the majority, the transcript size was consistent with our predictions (Table 1). In some cases (e.g., IgR#10, IgR#11 or IgR#13), multiple bands were observed. Two IgRs (#4 and #14) revealed a complex banding pattern (Table 1; Additional file 5) and further experiments are required to elucidate the origin of the detected RNA bands. Microarray analysis of strain 2011 RNA detected enrichment of RNA molecules < 200 nt corresponding to the predicted DNA regions for IgR#1, IgR#3, IgR#6, IgR#7, IgR#12, IgR#14, IgR#15, IgR#17 and IgR#18 (M-value > 2.5; Table 1). For the rest of the IgRs for which no signals were detected in Northern blot or microarray analysis, it may be that the transcript level is below our threshold of detection or that this candidate sRNA has a very specific inducing signal different from those included in our assays. This may be the case for IgR#5 with no detected bands in Northern blot (Figure 1) and a slightly lower enrichment detected in the microarray experiment (M-value = 2.15 under 45 min of saline stress; Table 2). In fact, two transcripts of different polarity (*sra12a *and *sra12b*) were reported for the same IgR in strain 1021 [27]. During the preparation of this manuscript, transcripts were reported in total RNA from strain 1021 for IgR#1, IgR#3, IgR#5, IgR#10, IgR#12 and IgR#13 [26,27]. Thus, our data independently confirmed the expression of those putative sRNAs under different experimental conditions and in a different but closely related strain, so we assume that the corresponding IgRs of strain 2011 encode the sRNA homologues of 6S RNA (*smrC22 *= *sra56*), *smrC9 *(= *sra32*), *sra12*, *sra25*, *smrC10 *(= *sra33*) and *smrC7 *(= *sra03*), respectively [26,27].

**Table 2:** Top 20 highest-scoring putative sRNA genes predicted by the global scoring procedure as independent transcriptional units in chromosomal IgRs of *S. meliloti *1021.

**Gene or ** **Designation^1^**	**IgR ** **length**	**Upstream ** **ORF**	**Orientation ** **Up sRNA Dn**			**Promoter^2^**	**Predicted ** **5'-end^3^**	**Predicted ** **3'-end^4^**	**Blast ** **score**	**Qrna ** **score**	**Global ** **score**	**Length ** **(nt)^5^**	**SP^6^**	**Microarray ** **detection^7^**
***smrC15 *(*sm3*)** ***smrC16 *(*sm3'*)**	652	SMc01226	< <	< <	< <	NN, s70 PhoB	1698732 1698968 *	1698618 1698820	6	2	98	114 151	y	<, C (1698954-1698905)

*sm4*	541	SMc01844	>	>	<	NN, s70	2371490, 2371606	2371852	1	1	82	256–362	n	>, S (2371745–2371696 2371735–2371784)

*sm5*	384	SMc03856	<	>	>	s70	3522121, 3522271	3522379	12	8	80	108–258	y	>, A, B, O, S (3522279–3522328)

*sm6*	491	SMc01202	<	<	<	NN, s70	1728153, 1728196, 1728269	1728021	1	1	62	120–248	n	-

***smrC14 *(*sm7*)** *sm7'*	922	SMc02051	< <	< <	< <	NN, s70 NN	1667614 1667983	1667488 1667769	3	5	58	126 214	n	<, S (1667516-1667467)

***sm8***	301	SMc04042	<	>	<	NN, s70	3046713	3046789	9	4	53	76	y	-

*sm9*	470	SMc02080	>	<	>	NN, s70	1635343, 1635411, 1635564	1635217	1	1	52	126–347	n	<, B, H, O (*) (1635305–1635354 1635259-1635210)

*sm10*	405	SMc00057	>	>	>	NN, s70	1091047, 1091106	1091343	1	1	52	237–296	n	-

***smrC9 *(*sm12*)**	710	SMc01933	<	<	>	NN, s70	1398427, 1398584	1398279	4	3	47	148–305	y	<, A, C, H, S (1398340–1398389 1398389-1398340)

***smrC7 *(*sm13*)**	472	SMc02850	<	>	>	NN, s70	201682	201829	4	3	47	147	y	-

*sm11*	1101	SMc01671	<	>	<	NN, s70	2475717, 2475862	2475961	5	2	47	99–244	y	-

*sm14*	410	SMc02139	<	>	>	NN, PhoB	573816, 573830 *	574040	4	1	45	210	y	-

***sra12 *(*sm17*)**	829	SMc02151	<	>	<	NN, s70	560780, 560916, 561000	561258	1	1	42	258–478	n	>, S (*) (560802–560753)

*sm16*	902	SMc02597	>	<	>	NN, s70	1198309, 1198440, 1198508	1198093	1	1	42	212–416	n	-

*sm18*	277	SMc01425	<	>	>	s70	2270996, 2271068	2271224	1	1	42	156–228	n	-

*sm23*	292	SMc01218	<	<	>	NN	1706863	1706715	1	3	34	148	n	-

*sm25*	396	SMc04289	<	>	>	s70, PhoB	2210185, 2210260 *	2210322	1	2	33	62–137	n	-

***sm26***	1270	SMc04453	<	<	>	NN	2321447	2321055	2	1	33	392	y	<, A, C, O, S (2321429–2321478)

*sm28*	826	SMc03014	>	<	>	NN, s70	713461, 713680, 713763	713290	1	1	32	170–463	n	-

*sm30*	644	SMc01257	<	>	>	NN, s70, PhoB	1518613, 1518748 *	1518988	1	1	32	234–375	n	-

^1 ^sRNA candidates reported in contemporary studies retain their original notation (*smrC*# or *sra*#) [26,27]. Otherwise, the designation *sm*# corresponds to the full list of sRNA predictions obtained in this work by the global scoring procedure (Additional file 17). Candidate IgRs are sorted according to its descending global score (GS). Bolded candidates have been verified experimentally ([26,27]; this work).

^2 ^NN, neural network promoter prediction; s70, σ^70^-dependent promoter; s54, RpoN (σ^54^)-dependent promoter promoter; PhoB, putative PhoB binding site.

^3 ^The coordinates of all putative promoters and/or transcription factor binding sites within an IgR are presented. The position given for NN is the 3' end of the identified sequence. For σ^70 ^the position given is 7 bases downstream of the 3' end of the -10 hexamer. The 3' end of the predicted binding site for σ^54 ^and PhoB is indicated with an asterisk.

^4 ^Given is the position of the last uridine at the end of the terminator sequence.

^5 ^Range of possible lengths based on putative 5' and 3' ends.

^6 ^y, candidate present in sRNAPredictHT search (Additional file 4); n, candidate absent in sRNAPredictHT output.

^7 ^As described in Table 1.

**Figure 2:**
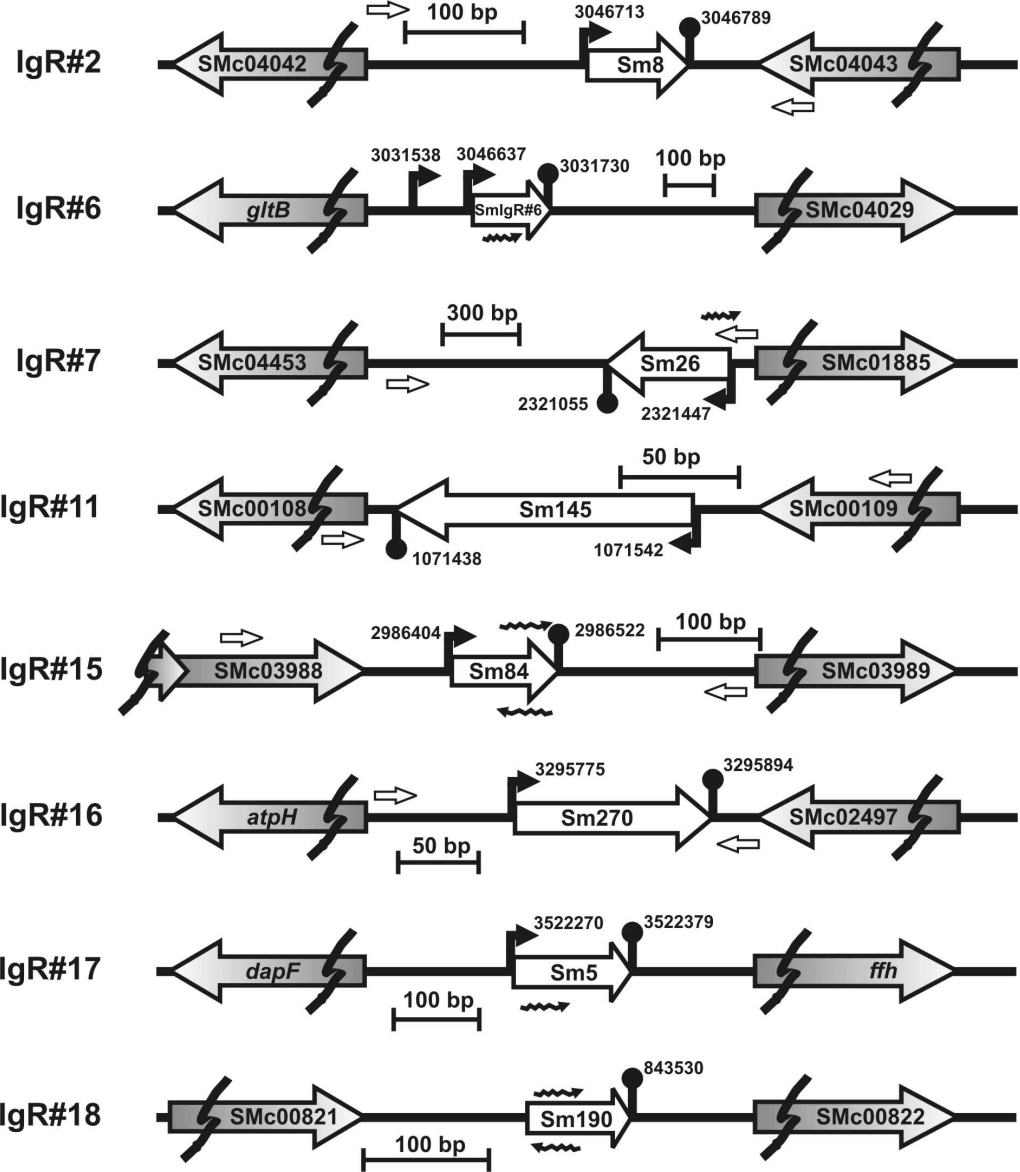
**Organization of novel *S. meliloti *1021 chromosomal loci encoding putative sRNAs with detected counterparts in *S. meliloti *strain 2011**. The IgRs encompassing novel putative sRNAs from our first compilation (Table 1) are drawn to scale in the portion between breaks. The chromosomal coordinates of predicted promoters and Rho-independent terminators are indicated next to the corresponding symbols. ORFs flanking each IgR are designated with their annotated codes or gene names. sRNAs are designated according to their position in the output of global scoring for the corresponding IgR (Additional file 17) or to the corresponding IgR from Table 1. Small empty arrowheads indicate the approximate position of the chromosomal target sequences for oligonucleotides used to generate probes for Northern blot. Wavy arrowheads denote the location and orientation of oligonucleotides present in Sm14kOLI microarray that detected the putative sRNA transcripts (Table 1).

To summarize, through this first compilation of putative sRNA-encoding IgRs, we obtained experimental evidence by Northern and/or microarray hybridization for eight novel *S. meliloti *RNA transcripts corresponding to candidates IgR#2, IgR#6, IgR#7, IgR#11, IgR#15, IgR#16, IgR#17 and IgR#18 (Table 1, Figure 1). Figure 2 shows the genomic context of these putative sRNA-encoding genes. The sequence alignments and associated transcriptional signals of these confirmed candidate loci are presented in Additional files 6, 7, 8, 9, 10, 11, 12, 13, 14, 15, 16.

### Growth phase and stress conditions influence accumulation of detected transcripts

For several of the sRNA transcripts detected by Northern analysis, we observed differential abundances under the various growth conditions tested (Figure 1). Transcripts originating from IgR#1, IgR#2, IgR#10 and IgR#13 were more abundant in stationary phase cells (> 2×), whereas those from IgR#7 and IgR#11 seemed to be downregulated in saturated cultures. The only RNA species that was clearly upregulated under high salt conditions was the one coded in IgR#11 (> 6×). Agents that alter membrane fluidity, as SDS or ethanol, induced accumulation (> 2×) of transcripts from IgR#1, IgR#2, IgR#7, IgR#10, IgR#11 and IgR#13. In *E. coli*, several sRNAs participate in the control of porin levels upon membrane stress [9]. An increase in growth temperature from 28 to 37°C resulted in upregulation (> 2×) of transcripts from IgR#2 and IgR#11. Upon phosphate starvation, the transcripts from IgR#1, IgR#7, IgR#15 and IgR#16 were upregulated. A conserved PhoB binding site [51] is not evident upstream the predicted promoter for these sRNA candidates, suggesting that the positive regulation may be indirect or PhoB-independent. Finally, exposure of *S. meliloti *2011 to pH 5.5 for 90 minutes, an acid stress condition that does not support growth [60], resulted in accumulation of RNAs from IgR#1, IgR#3, IgR#10 and IgR#13. For IgR#6, IgR#12, IgR#17 and IgR#18, for which no Northern hybridization data was available, we could observe an enrichment of short transcripts upon 45 min of stress conditions using microarray analysis (Table 1).

The observed expression pattern for IgR#1 is consistent with that observed for 6S RNA homologues in other bacteria. The transcript accumulated in stationary phase cells, in the presence of SDS, under phosphate deprivation and more markedly under conditions of acid stress. The level of 6S RNA increases along the growth curve being maximal in stationary phase in *E. coli *[61] and *B. subtilis *cells [62]. This correlates with a reduced utilization of the vegetative σ^70 ^subunit by the RNA polymerase complex in favour of alternative sigma subunits [63,64]. The abundance of the sRNA from IgR#2 detected in strain 2011 was upregulated both in response to increased cell density as well as to several different stress conditions (Figure 1). This sRNA had previously been annotated as SuhB [65] but had not been subjected to experimental verification.

While the abundance of a significant number of the sRNAs identified in this study appears to be significantly affected by growth phase and/or environmental stress conditions (Figure 1, Table 1), it is still unclear how this regulation is effected. Conserved sequences suggestive of upstream regulatory sites were not detected for any of the sRNA loci confirmed in this study (Additional files 6, 7, 8, 9, 10, 11, 12, 13, 14, 15, 16). Time course studies of transcript levels upon stress application together with the study of promoter expression *in vitro *and *in planta *are currently being undertaken to elucidate the regulatory mechanisms underlying the observed differences in transcript abundance. Moreover, strains deleted for or overexpressing these sRNAs are being constructed in an effort to gain insights into the biological functions of these sRNAs both during *S. meliloti *growth in culture and during its symbiotic interaction with the host plant.

### Improvement of the bioinformatic predictive method and application to *S. meliloti *chromosome

**Figure 3:**
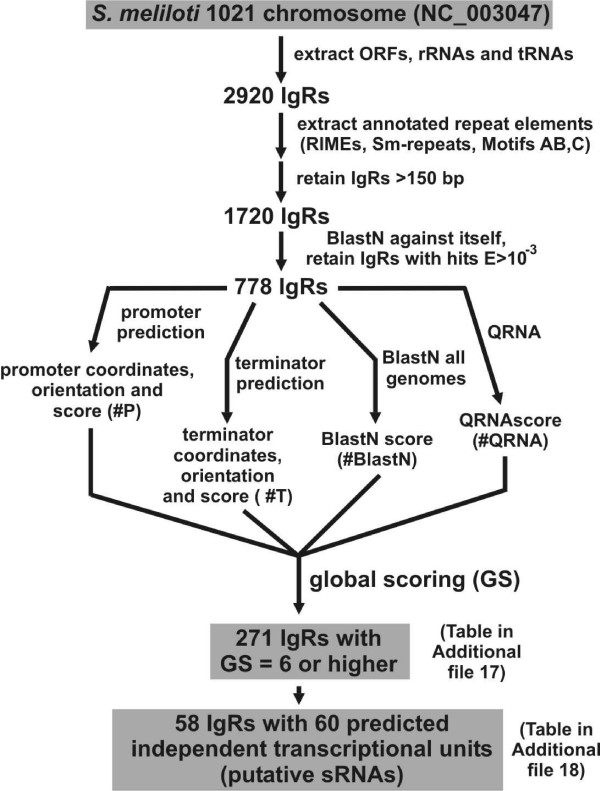
**Improvement of the predictive strategy of chromosomal *S. meliloti *sRNAs**. From the initial list of 2920 chromosomal IgRs, we retained 778 IgRs longer than 150 bp than did not contain annotated or non-annotated repeats. Next, we introduced a global scoring criterion for each IgR to assign a numerical score taking into account the presence of putative independent transcriptional units or transcriptional signals and their relative distance to flanking ORFs, sequence conservation (BlastN analysis) and secondary structure conservation (QRNA analysis). See text for further details.

**Figure 4:**
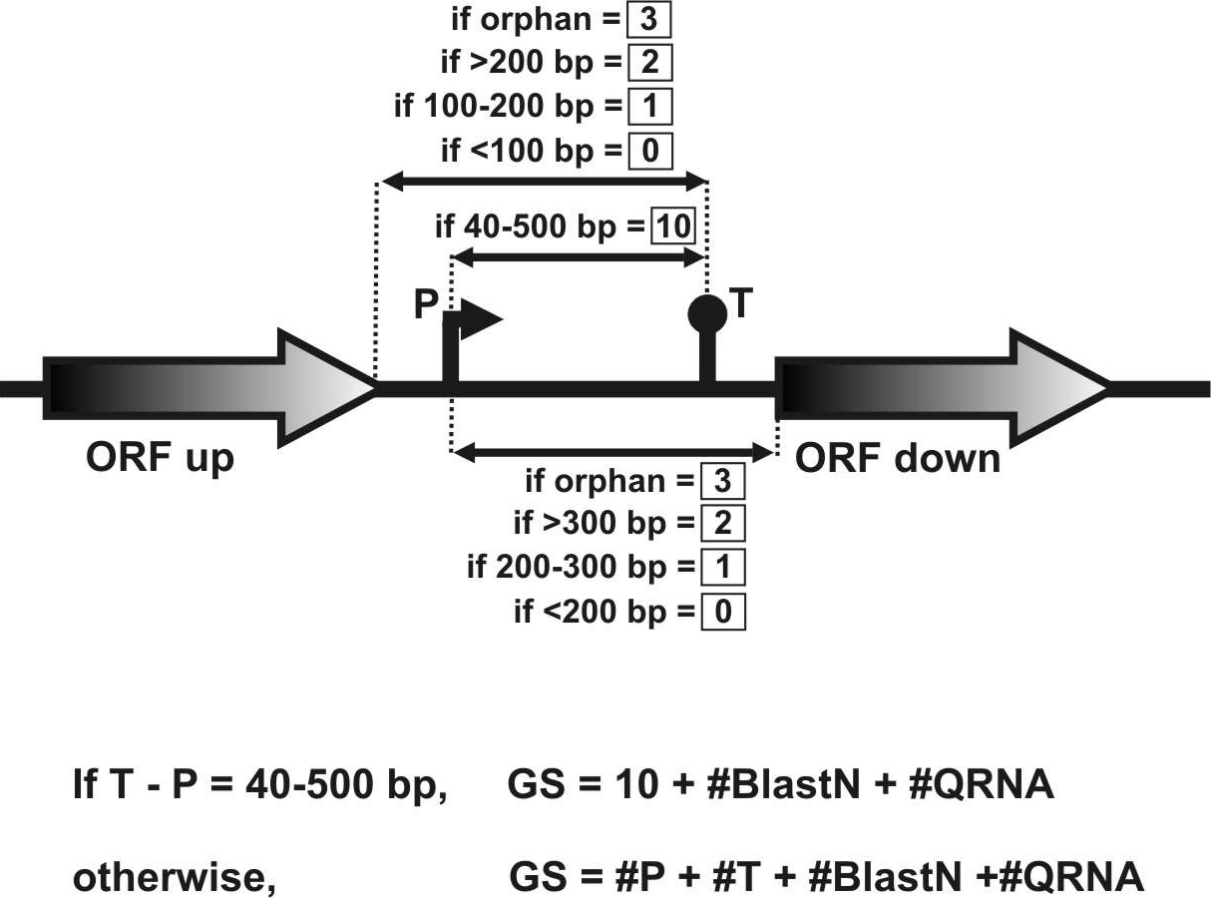
**Summary of the scoring criteria introduced to weigh the relative position of predicted transcriptional signals in IgRs**. An IgR with a co-oriented putative promoter and a terminator separated from by 40–500 bp each other was scored 10. Every promoter-terminator pair matching the previous criterion within a single IgR was rated individually and summed to calculate the global score of that IgR. Orphan promoters were scored 3. Putative promoters were rated 2 if the 5'-end of the co-oriented flanking CDS was farther than 300 bp, 1 if this distance was 200–300 bp and 0, if they were closer than 200 bp. Orphan terminators were scored 3. Predicted terminators co-oriented with flanking ORFs were scored according to their relative distance to the 3'-end of the corresponding annotated gene, so that a score of 2 was assigned if the terminator was farther than 200 bp, 1 if the distance was 100–200 nt, and 0 if it was closer than 100 bp.

Our initial computational screen proved quite accurate in identifying both previously identified and novel sRNAs (Table 1, Figures 1 and 2). However, the parameters used in this screen were quite stringent, requiring nearly all candidate loci to be associated with a putative promoter, a predicted terminator, and conserved intergenic sequence. We therefore postulated that a significant number of *S. meliloti *sRNA-encoding genes were likely missed using our initial predictive approach. To increase the sensitivity of our computational screen, we modified our predictive algorithm so that sRNA-encoding genes are identified based on their association with any or all of the following predictive features: transcriptional terminators, promoters, primary sequence conservation, and secondary structure conservation. Bioinformatic searches using similar algorithms [13,66] have often yielded a large proportion of false predictions, significantly decreasing the efficiency in which putative sRNA loci could be experimentally confirmed. Based on these previous studies, we were concerned that increasing the sensitivity of our predictive approach would result in a significant decrease in its accuracy. To address this concern, we incorporated a novel scoring algorithm that allows predicted loci to be ranked based on their likelihood of encoding a *bona fide *sRNAs (Figures 3 and 4). This allows stronger candidates to be readily identified and prioritized for experimental verification and characterization.

In our improved computational approach, IgRs are analysed for the presence of transcriptional signals (promoters and terminators), sequence conservation (BlastN) and secondary structure conservation (QRNA) (Figure 3), and receive a corresponding score for each item (Figure 4). Thus, prediction of a promoter and a terminator co-oriented but separated for > 40 bp and < 500 bp, determines a score = 10 for this pair of signals for a given IgR (Figure 4). Instead, if only one of the signals is present (terminator or promoter) or both are predicted but not co-oriented, the maximum score for each signal would be 3 (Figure 4). Similarly, a score is assigned to each IgR based on the presence of sequence and/or secondary structure conservation (see Methods). These different scoring analyses are integrated by the assignment of a global score (GS) calculated as the sum of the individual scores (Figure 4).

We first applied our improved predictive approach to the *S. meliloti *1021 chromosome (Figure 3 illustrates the pipeline for the chromosome). We limited our searches to IgRs 150 bp or longer that do not contain annotated or non-annotated repetitive sequences. We found that *S. meliloti *IgRs containing experimentally verified sRNA transcripts were assigned a GS of 6 (IgR#16) or higher (up to GS = 168, for the RNAse P RNA) ([26,27]; Table 3); thus we established GS = 6 as the cut-off for sRNA prediction. Our predictive scheme identified and ranked 271 IgRs with GS ≥ 6 (Additional file 17). We designated the candidate RNA elements as *sm*# (*sm1 *to *sm271*). SsrA, RNAse P RNA, 4.5S RNA and 6S RNA were ranked within the top 32 hits (Additional file 17). All 18 of the IgRs initially selected for experimental verification (Table 1) are contained within the list of candidate sRNA genes (Additional file 17). From the entire set, we extracted a subset of 58 IgRs predicted to contain 60 transcriptional units (i.e., a predicted promoter co-oriented with a predicted terminator separated at 40–500 bp; Figure 4) (Additional file 18). Eleven of the 18 IgRs initially compiled were included in the subset of predicted transcriptional units; the other 7 IgRs were missing from this subset because either they lack typical transcription signatures (IgR#1, IgR#4 and IgR#18; Table 1) or they differ significantly from the queried consensus and only became evident as conserved regions in sequence alignments with IgRs of related α-proteobacteria (IgR#6, IgR#10, IgR#12 and IgR#16; Table 1). On the other hand, 42 of the 60 listed candidate transcriptional units in this list (Additional file 18) had not been identified previously by sRNAPredictHT (Additional file 4).

**Table 3:** Other small RNAs and cis-regulatory RNA elements detected in the chromosome of *S. meliloti *1021 by the global scoring procedure.

**Prediction**	**Rfam^1^**	**IgR coordinates**	**upstream ORF^2^**	**RNA element^2^**	**downstream ORF^2^**	**GS^3^**	**Ref.**
RNAseP RNA	y	2356367–2357249	SMc01857<	<	SMc01856<	168	[73]
FMN riboswitch	y	2398059–2398373	SMc01608>	>	*ribH2 *(SMc01609)>	127	[67]
4.5S RNA (SRP)	y	259823–260123	SMc02904<	>	*dnaX*>	41	[67]
Glycine riboswitch	y	1674762–1675635	*gcvT<*	<	SMc01242>	37	[67]
*ssrA *(tmRNA)	n	2290955–2291142	SMc01449<	>	SMc01450>	33	[23]
*smrC45 *(*speF*)	y	3105052–3105637	SMc02983<	<	SMc02984>	27	[65]
Thiamine riboswitch	y	3532759–3533026	SMc03868<	>	SMc03869>	19	[67]
*ilvIH *cis regulatory	n	2276530-2276876	SMc01431 (*ilvIH*)<	<	SMc01432>	16	[74]
Cobalamin riboswitch	y	1999510–1999806	SMc00166<	<	SMc00165<	16	[67]
Cobalamin riboswitch	n	2122378–2123250	SMc04305 (*cobPW*)<	<	SMc04306>	14	[67]
Cobalamin riboswitch	y	954440–955076	SMc00983<	>	SMc00982>	11	[67]
*ybhL *cis regulatory	y	3505880–3506125	SMc03838>	>	SMc03839>	3	[65]
*serC *cis regulatory	y	2938993–2939230	*serC*<	<	SMc00639<	2	[65]
Methionine riboswitch	y	3461713–3461838	SMc03796>	>	*metA*>	-	[65]
Methionine riboswitch	y	580068–580264	*metZ*<	<	SMc02218>	-	[65]

^1 ^RNA gene or element present (y) or absent (n) in Rfam database.

^2 ^The transcribed strand is indicated.

^3 ^global score (GS) calculated for the element in our predictive procedure (Figure 3).

The 20 top-scoring candidate IgRs with predicted transcriptional units consistent with putative sRNAs are listed in Table 2. For 8 of these IgRs we observed microarray signals from exponential phase cells of strain 2011 RNA upon introduction of various stress conditions (Table 2). Thus, there is experimental evidence to date for 10 candidate sRNA loci among those top 20 IgRs (Table 2); i.e. *smrC15 *and *smrC16 *[26] (= *sra41*; [27]), *sm4 *(this work), *sm5 *(this work), *smrC14 *[26] (= *sm7*; this work), *sm8 *(this work), *sm9 *(this work), *smrC9 *[26] (= *sra32*; [27], = *sm12*; this work), *smrC7 *[26] (= *sra03*; [27], = *sm13*; this work), *sra12 *[27] (= *sm17*; this work) and *sm26 *(this work). The high proportion of confirmed sRNAs among these high-scoring loci suggests many of the 10 still unidentified candidates in this cohort correspond to *bona fide *sRNA-encoding genes. Another remarkable feature of our predictive method is that it was able to locate quite precisely the limits of the transcriptional units. The predicted transcription start site and the last uridine of *smrC15 *(*sm3 *in Table 2), *smrC14 *(*sm7 *in Table 2), *smrC9 *(*sm12 *in Table 2) and *smrC7 *(*sm13 *in Table 2), differ by only 1–3 bp from the experimentally determined 5'- and 3'-termini [26]. This is also an important validation of the *in silico *prediction of IgR transcriptional units. It is noteworthy that the IgR with the highest global score is predicted to encode two independent sRNAs (Table 2). This region has recently been shown to encode two sRNA loci, *smrC15 *and *smrC16 *[26], with remarkably similar sizes to those predicted in this study (*sm3 *and *sm3'*). Another IgR is predicted to encode two independent sRNAs (*sm7 *and *sm7'*; Table 2) one of which has been experimentally verified (*smrC14 *= *sm7*) and the second of which awaits experimental detection [26].

Our predictive method also identified sequence elements that correspond to highly conserved small non-coding RNAs (Table 3). These predictions include the RNAseP RNA component (GS = 168), the SRP RNA component (4.5S RNA; GS = 41), *ssrA *(tmRNA; GS = 33) and 6S RNA (GS = 27) [23,27,67], as well as cis-regulatory RNA elements as the FMN (GS = 127), glycine (GS = 37), thiamine (GS = 19), and cobalamin (GS 11–16) riboswitches and other putative cis-acting motifs as the *ilvIH *5' trailer (GS = 16). One particular interesting locus is the α-proteobacterial *speF *element (GS = 27; Table 3) that was first described as a possible 5'-UTR regulatory element associated with an ornithine decarboxylase mRNA gene [65,67], but was recently detected as a candidate sRNA that may be independently transcribed (*smrC45*; [26]).

### Putative sRNA-encoding genes in *S. meliloti *megaplasmids

We next applied our improved predictive approach to the *S. meliloti *1021 megaplasmids pSymA and pSymB. The list of independent transcriptional units that may represent novel sRNA-encoding genes in pSymA and pSymB is presented in Table 4. A significantly lower density of independent transcriptional units was identified in megaplasmids (an average of 8–9 sRNA genes per Mb) than in the chromosome (*ca*. 16 sRNA genes per Mb). It is noteworthy that most of the candidate transcripts were validated in our microarray screen (Table 4), strongly supporting the predictive methodology. Most of the transcripts were enriched > 5.5-fold (M-value > 2.5) in RNA from strain 2011 subjected to various stress stimuli, and two others (*smA1 *and *smB7*) showed a > 4-fold induction (M-value > 2) (Table 4).

**Table 4:** Putative sRNA genes predicted as independent transcriptional units in IgRs of *S. meliloti *1021 megaplasmids pSymA (*smA*#) and pSymB (*smB*#).

**Gene or ** **Designation^1^**	**IgR ** **length**	**Upstream ** **ORF**	**Orientation ** **Up sRNA Dn**			**Promoter^2^**	**Predicted ** **5'-end^3^**	**Predicted ** **3'-end^4^**	**Length ** **(nt)^5^**	**Microarray ** **detection^6^**
*smA1*	719	SMa0450	>	>	>	s70	241667	241961	294	>, C, O (*) (241991-241942)

*smA2a* *smA2b* *smA2c*	817	SMa0585	< < <	< < >	> > <	s54 s70 NN	313672 * 313772 313667	313529 313529 314181	143–243 444	>, A, B, C, H, S (314210-314161 313991–314040 314162–314211 313784–313833)

*smA3a* *smA3b*	1007	SMa0922	>	<	<	NN s70	512362 512439	512154	208–285	<, A, C (512371–512420)

*smA4a* *smA4b*	714	SMa0995	< <	> >	> >	NN NN	552600 552853	552980	127–280	>, A, B, C, H, O, S (552880–552929 552801-552752 552954-552905)

*smA5*	292	SMa1024	<	>	>	PhoB	567336 *	567465	129	>, H, S (567397-567348)

*smA6*	624	SMa1245	<	<	<	NN	682304	682237	67	<, O (682278-682229)

*smA7*	879	SMa1644	> >	> >	< <	s70 NN, s70	916928 917145	917238	93–310	-

*smA8*	1124	SMa2165	>	>	>	NN	1220442	1220807	365	>, A, H, O, S (1220782–1220831)

*smB1*	282	SMb20203	>	>	>	NN	213506	213617	111	-

*smB2*	495	SMb20366	>	>	<	NN	379143	379269	126	>, H, S (379180–379131)

*smB3a* *smB3b* *smB3c*	750	SMb20516	> > >	< < <	< < <	NN NN s70	541963 541911 541833	541769	64–194	<, H (541835–541884)

*smB4a* *smB4b*	592	SMb20543	<	<	<	NN, s70 s70	571374 571340	571217	123–157	<, A, C (571271-571222 571222–571271)

*smB5a* *smB5b*	1623	SMb20548	> >	> >	> >	NN NN, s70	574477 574621	574763	142–286	>, A, sB, C, H, O, S (574671-574622)

*smrB35 *(*smB6*)	751	SMb20551	<	>	>	s70	577731	577869	138	>, O, S (577781-577732)

*smB7*	196	SMb21316	<	>	>	s70	983786	983878	92	>, S (*) (983845-983796)

*smB8*	767	SMb20872	<	<	<	NN, s70 s70	1279964 1281110	1279836	128–274	<, A, O (1279979–1280028 1280182-1280231)

*smB9*	1014	SMb20916	<	>	>	s70	1325140	1325588	448	>, H (1325459–1325508)

*smB10*	506	SMb20748	<	<	>	NN	1537794	1537673	121	-

^1 ^sRNA candidates verified experimentally retain their original notation [26].

^2 ^NN, neural network promoter prediction; s70, σ^70^-dependent promoter; s54, RpoN (σ^54^)-dependent promoter promoter; PhoB, putative PhoB binding site.

^3 ^The coordinates of all putative promoters and/or transcription factor binding sites within an IgR are presented. The position given for NN is the 3' end of the identified sequence. For σ^70 ^the position given is 7 bases downstream of the 3' end of the -10 hexamer. The 3' end of the predicted binding site for σ^54 ^and PhoB is indicated with an asterisk.

^4 ^Given is the position of the last uridine at the end of the terminator sequence.

^5 ^Range of possible lengths based on putative 5' and 3' ends.

^6 ^As described in Table 1.

One of the candidates identified in pSymB and detected in strain 2011 (*smB5b*; Table 4) seems to be a second copy of the strain 1021 transcript *smrB35 *[26] (= *smB6*; Table 4). Interestingly, the plasmid-encoded candidate *smB5b *that lies in an IgR only 3 kb upstream *smrB35 *was found to share 64% sequence identity with *smrB35 *(= *smB6*) (Additional file 19). Finally, the pSymA candidate *smA4b *was found to share sequence similarity with both chromosomally-encoded candidates *smrC15 *and *smrC16 *[26] (60% and 67%, respectively; Additional file 19). The putative sRNA *smA4b *is encoded within an IgR flanked by two transposable elements, ISRm5 and ISRm25, both containing their corresponding transposase genes SMa0995 and SMa0997 (Additional file 19), suggesting *smA4b *may have been acquired through horizontal transfer. The incompatibility-related pSymA and pSymB *incA *sRNAs were not detected as transcriptional units because they bear atypical terminators [25], but their σ^70^-dependent promoters were precisely predicted (data not shown).

### Comments on the global scoring procedure

Our findings suggest that the GS method is effective in identifying both known and novel sRNA genes. Half of the chromosomal and 80% of the megaplasmid IgRs predicted to contain transcriptional units suggestive of sRNA genes have been validated experimentally in this (Tables 2 and 4) and other works [26,27]. However, it is yet unclear what proportion of all candidate predictions correspond to false positives. On the other hand, the number of potential sRNA loci may be underestimated in this study as we have ignored protein coding DNA sequences as a source of sRNA transcripts [56,57,68]. Another underestimation comes from the possibility that certain sRNA genes may require RNA polymerase sigma factors different from those screened here or may have atypical terminator sequences (as the aforementioned *incA *genes from pSymA and pSymB). As consensus sequences for additional *S. meliloti *transcription factors are determined, the modular design of our predictive protocol will allow these motifs to be readily incorporated into future searches. It is important to note that our method does not exclude the possibility that the identified putative sRNA loci, if expressed, do translate into short peptides [69] or are integral parts of mRNAs such as 5'-UTR leader regions. In fact, there are reports of sRNAs that are generated by post-transcriptional processing of mRNAs [70] including self-cleavage of riboswitch elements [71]. Our systematic procedure for IgR sorting is largely dependent on the utilization of open source tools (e.g., TranstermHP, Fuzznuc, Rsat, NNPP, BlastN, Rfam, QRNA) Thus, this methodology could be readily applied to any annotated DNA sequence for which appropriate BLAST partners and promoter consensus sequences are available. One key feature of our GS methodology is that the relative weighing of individual scores for transcriptional signatures and conservation features may be modified to generate different priority listings of candidate IgRs. We therefore anticipate that our predictive approach can be flexibly implemented in identifying sRNAs in many other bacterial species.

## Conclusion

We have utilized the chromosomal DNA information of the sequenced strain *S. meliloti *1021 to compile a first list of candidate sRNA genes (Table 1). By a combination of Northern hybridization and microarray analysis of RNA from the highly similar strain 2011, we here report eight novel sRNA loci (Table 1, Figures 1 and 2). Significant variation of transcript abundance was observed for many of the confirmed sRNAs of our first compilation under different growth conditions (Figure 1 and Table 1), providing important clues into their regulation and potential regulatory function.

The experimentally verified non-coding RNAs of *S. meliloti*, other than *ssrA*, 4.5S RNA, 6S RNA and RNAse P RNA ([26,27], this work), may encode regulatory sRNAs of the base-pairing mechanism. Two lines of sequence-based evidence suggest that *S. meliloti *and probably other α-proteobacteria as well, does not encode sRNAs of the molecular mimic type. First, no homologues of the translational regulator RNA-binding proteins of the RsmA/CsrA family could be detected by aminoacid sequence similarity (PSI-BLAST) in α-proteobacteria. Second, when we applied the CSRNA_FIND algorithm [72] to *S. meliloti *1021 chromosomal and pSym IgRs, it did not detect a significantly higher density over the average of A(R)GGA sequence motifs, the hallmark of the molecular mimic sRNAs of the RsmZ/CsrB family [8]. Thus, most likely, *S. meliloti *only contains regulatory sRNA genes of the antisense trans-acting type [4].

To identify additional *S. meliloti *sRNA genes, we conducted a bioinformatic screen with a novel algorithm designed to more sensitively detect previously unannotated genes. The results of these screens significantly expand the list of putative sRNA-encoding IgRs in the three replicons of *S. meliloti *1021 and in closely related α-proteobacteria. Importantly, microarray data provided a strong support to our GS approach for prediction of putative sRNA-encoding genes (Tables 2 and 4). One advantage of the scoring criterion used here is that allows the strongest candidate loci to be prioritized for experimental verification and characterization. Thus, as bioinformatic screens continue to identify putative sRNA-encoding genes at rates that far exceed the throughput of existing experimental tools for sRNA confirmation and characterization, this prioritization of candidate genes should be very helpful in confirming and unravelling the diverse biological roles of these important and ubiquitous riboregulators.

## Authors' contributions

CV conceived of the study, coordinated the research, carried out Northern blot analysis and wrote the manuscript; JL executed the bioinformatics search with sRNApredictHT; JPS carried out microarray hybridization experiments and microarray data analysis; JR contributed to the analysis of microarray data; AB designed the transcriptomic experiments and produced the microarrays; GP participated in the design, programming and execution of the Global Score algorithm. All authors revised and approved the final version of the manuscript.

## Supplementary Material

Additional file 1 **Crude sRNApredictHT predictions on *S. meliloti *1021 genome.** Direct output of the sRNAPredictHT algorithm applied to the complete set of *S. meliloti *1021 chromosomal IgRs. Click here for file

Additional file 2 **Oligonucleotides used for synthesis of Northern blot probes.** Oligonucleotides used to PCR amplify the IgR sequences encompassing sRNA candidates. Click here for file

Additional file 3 **Denaturing PAGE fractionation of *S. meliloti *2011 total RNA.** Electrophoretic pattern of *S. meliloti *2011 total RNA in a denaturing polyacrylamide gel (8.3 M urea, 8% acrylamide and 0.2% bisacrylamide in 1× TBE buffer; 25 cm-long). Approximately 20–60 μg of total RNA, corresponding to all cells present in 20 ml of RDM cultures, were loaded in each lane. The gel was stained with ethidium bromide and visualized on an UV transilluminator. Under this conditions, effective fractionation of RNAs < 600 nt was achieved. RNA bands of varying intensity in different samples are indicated with arrowheads. Stat, stationary phase cells; log, exponential phase cells; NaCl 0.3 M, saline stress, H_2_O_2_, oxidative stress; pH 4.0, acid stress; SDS and EtOH, membrane stress; -P, phosphate starvation; 0°C, cold shock; 45°C, heat shock. Click here for file

Additional file 4 **Curated sRNApredictHT predictions. **The sRNAPredictHT output listed in Additional file 1 was curated upon elimination of IgRs containing annotated and non annotated repeats. Click here for file

Additional file 5 **Expression of putative sRNAs in IgR#4 and IgR#14.** Northern blot analysis of putative sRNAs encoded in IgR#4 and IgR#14. See legend to Figure 1 for details. Click here for file

Additional file 6 **Novel candidate sRNA gene *sm8 *in IgR#2.** Conservation of the novel candidate sRNA gene *sm8 *(IgR#2) in α-proteobacteria. Sequence alignment generated with ClustalW for the corresponding IgRs of *S. meliloti *1021 (1021), *Sinorhizobium medicae *WSM419 (Smed), *Agrobacterium tumenfaciens *C58 (At), *Rhizobium etli *CFN42 (Retli) and *Rhizobium leguminosarum *bv *viciae *3841 (Rleg). The Rho-independent terminator was predicted for *S. meliloti 1021 *(see text) and confirmed from conserved positions in the alignment. The putative sigma 70-dependent promoter (-10 and -35 hexamers) and transcription start site (+1) were deduced from conserved positions in the alignment. The secondary structure presented for *S. meliloti *Sm8 RNA was calculated with the Mfold server [75] and corresponds to the predicted structure with lower free energy. Click here for file

Additional file 7 **Novel candidate sRNA gene *sm137 *in IgR#4.** Conservation of the novel candidate sRNA gene *sm137 *(IgR#4) in α-proteobacteria. Sequence alignment generated with ClustalW for the corresponding IgRs of *S. meliloti *1021 (1021), *S. medicae *WSM419 (Smed), *R. etli *CFN42 (Retli) and *R. leguminosarum *bv *viciae *3841 (Rleg). The Rho-independent terminator was predicted for *S. meliloti 1021 *(see text) and confirmed from conserved positions in the alignment, but there was no prediction of a promoter in this IgR. Click here for file

Additional file 8 **Novel candidate sRNA gene *sm*IgR#6.** Conservation of the novel candidate sRNA gene *sm*IgR#6 in α-proteobacteria. Sequence alignment generated with ClustalW for the corresponding IgRs of *S. meliloti *1021 (1021), *R. etli *CFN42 (Retli) and *R. leguminosarum *bv *viciae *3841 (Rleg). Two putative sigma 70-dependent promoters (-10 and -35 hexamers), transcription start sites (+1) and a single Rho-independent terminator were predicted for *S. meliloti 1021 *(see text). The secondary structure presented for both possible *S. meliloti *sRNAs from IgR#6 were calculated with the Mfold server [75] and correspond to the predicted structures with lower free energy. Click here for file

Additional file 9 **Novel candidate sRNA gene *sm26 *in IgR#7.** Conservation of the novel candidate sRNA gene *sm26 *(IgR#7) in α-proteobacteria. Sequence alignment generated with ClustalW for the corresponding IgRs of *S. meliloti *1021 (1021), *S. medicae *WSM419 (Smed), *R. etli *CFN42 (Retli) and *R. leguminosarum *bv *viciae *3841 (Rleg). The putative sigma 70-dependent promoter (-10 and -35 hexamers), transcription start site (+1) and Rho-independent terminator were predicted for *S. meliloti 1021 *(see text). The secondary structure presented for *S. meliloti *Sm26 RNA was calculated with the Mfold server [75] and corresponds to the predicted structure with lower free energy. Click here for file

Additional file 10 **Candidate sRNA gene *sm64 *(*sra25*) in IgR#10.** Conservation of the candidate sRNA gene *sm64 *(IgR#10; *sra25*, [27]) in α-proteobacteria. Sequence alignment generated with ClustalW for the corresponding IgRs of *S. meliloti *1021 (1021), *S. medicae *WSM419 (Smed), *R. etli *CFN42 (Retli) and *R. leguminosarum *bv *viciae *3841 (Rleg). The putative sigma 70-dependent promoter (-10 and -35 hexamers), transcription start site (+1) and Rho-independent terminator were predicted for *S. meliloti 1021 *(see text) and confirmed from conserved positions in the alignment. The secondary structure presented for *S. meliloti *Sm64 RNA was calculated with the Mfold server [75] and corresponds to the predicted structure with lower free energy. Click here for file

Additional file 11 **Novel candidate sRNA gene *sm145 *in IgR#11.** Conservation of the novel candidate sRNA gene *sm145 *(IgR#11) in α-proteobacteria. Sequence alignment generated with ClustalW for the corresponding IgRs of *S. meliloti *1021 (1021), *S. medicae *WSM419 (Smed), *A. tumenfaciens *C58 (At), *R. etli *CFN42 (Retli) and *R. leguminosarum *bv *viciae *3841 (Rleg). The putative sigma 70-dependent promoter (-10 and -35 hexamers), transcription start site (+1) and Rho-independent terminator were predicted for *S. meliloti 1021 *(see text) and confirmed from conserved positions in the alignment. The secondary structure presented for *S. meliloti *Sm145 RNA was calculated with the Mfold server [75] and corresponds to the predicted structure with lower free energy. Click here for file

Additional file 12 **Novel candidate sRNA gene *sm76 *in IgR#14.** Conservation of the novel candidate sRNA gene *sm76 *(IgR#14) in α-proteobacteria. Sequence alignment generated with ClustalW for the corresponding IgRs of *S. meliloti *1021 (1021), *S. medicae *WSM419 (Smed), *A. tumenfaciens *C58 (At), *R. etli *CFN42 (Retli), *R. leguminosarum *bv *viciae *3841 (Rleg), *Mesorhizobium loti *MAFF303099 (Mloti), *Ochrobactrum anthropi *ATCC49188 (Oa) and *Brucella ovis *ATCC25840 (Bo). The putative sigma 70-dependent promoter (-10 and -35 hexamers), transcription start site (+1) and Rho-independent terminator were predicted for *S. meliloti 1021 *(see text) and confirmed from conserved positions in the alignment. A second putative promoter was predicted for *S. meliloti *upstream then conserved one, but it seems to be specific for *Sinorhizobium*. The alternative secondary structures presented for *S. meliloti *Sm76 RNA were calculated with the Mfold server [75] and corresponds to the predicted structure with lower free energy for the two possible transcripts. Click here for file

Additional file 13 **Novel candidate sRNA gene *sm84 *in IgR#15.** Conservation of the novel candidate sRNA gene *sm84 *(IgR# 15) in α-proteobacteria. Sequence alignment generated with ClustalW for the corresponding IgRs of *S. meliloti *1021 (1021), *S. medicae *WSM419 (Smed), *A. tumenfaciens *C58 (At), *R. etli *CFN42 (Retli) and *R. leguminosarum *bv *viciae *3841 (Rleg). The putative sigma 70-dependent promoter (-10 and -35 hexamers), transcription start site (+1) and Rho-independent terminator were predicted for *S. meliloti 1021 *(see text) and confirmed from conserved positions in the alignment. The secondary structure presented for *S. meliloti *Sm84 RNA was calculated with the Mfold server [75] and corresponds to the predicted structure with lower free energy. Click here for file

Additional file 14 **Novel candidate sRNA gene *sm270 *in IgR#16.** Conservation of the novel candidate sRNA gene *sm270 *(IgR#16) in α-proteobacteria. Sequence alignment generated with ClustalW for the corresponding IgRs of *S. meliloti *1021 (1021), *S. medicae *WSM419 (Smed), *A. tumenfaciens *C58 (At), *R. etli *CFN42 (Retli) and *R. leguminosarum *bv *viciae *3841 (Rleg). The putative sigma 70-dependent promoter (-10 and -35 hexamers), transcription start site (+1) and the putative Rho-independent terminator were predicted for *S. meliloti 1021 *(see text) and confirmed from conserved positions in the alignment. The secondary structure presented for *S. meliloti *Sm270 RNA was calculated with the Mfold server [75] and corresponds to the predicted structure with lower free energy. Click here for file

Additional file 15 **Novel candidate sRNA gene *sm5 *in IgR#17.** Conservation of the novel candidate sRNA gene *sm5 *(IgR#17) in α-proteobacteria. Sequence alignment generated with ClustalW for the corresponding IgRs of *S. meliloti *1021 (1021), *R. etli *CFN42 (Retli) and *R. leguminosarum *bv *viciae *3841 (Rleg). The putative sigma 70-dependent promoter (-10 and -35 hexamers), transcription start site (+1) and the putative Rho-independent terminator were predicted for *S. meliloti 1021 *(see text) and confirmed from conserved positions in the alignment. The secondary structure presented for *S. meliloti *Sm5 RNA was calculated with the Mfold server [75] and corresponds to the predicted structure with lower free energy. Click here for file

Additional file 16 **Novel candidate sRNA gene *sm190 *in IgR#18.** Conservation of the novel candidate sRNA gene *sm190 *(IgR#18) in α-proteobacteria. Sequence alignment generated with ClustalW for the corresponding IgRs of *S. meliloti *1021 (1021), *R. etli *CFN42 (Retli) and *R. leguminosarum *bv *viciae *3841 (Rleg). The putative Rho-independent terminator was predicted for *S. meliloti 1021 *(see text) and confirmed from conserved positions in the alignment. The secondary structure presented for *S. meliloti *Sm190 RNA was calculated with the Mfold server [75] and corresponds to the predicted structure with lower free energy assuming that the sRNA extends along the conserved sequence upstream the terminator and includes the terminator itself. Click here for file

Additional file 17 **List of *S. meliloti *sRNA gene predictions based on the GS method.** Complete list of *S. meliloti *1021 chromosomal IgRs predicted to encode sRNAs according to the global scoring procedure (summarized in Figure 3). Click here for file

Additional file 18 **Predicted transcriptional units in *S. meliloti *1021 chromosomal IgRs.** sRNA candidates identified as putative transcriptional units in intergenic regions of *S. meliloti *1021 chromosome. Click here for file

Additional file 19 **Homologs of sRNA genes *smrC14*-*smrC15 *and *smrB35 *are present in pSymA and pSymB IgRs.** Identification of extra copies of sRNA genes *smrC14*-*smrC15 *and *smrB35 *in pSymA and pSymB IgRs. Genetic surroundings and sequence alignments generated with ClustalW for the sRNA genes of *S. meliloti *1021 *smrC14*, *smrC15 *[26] and the corresponding identified homolog in pSymA (*smA4b*; Table 4), and for the sRNA gene *smrB35 *[26] and the corresponding identified homolog in pSymB (*smB5b*; Table 4). The putative sigma 70-dependent promoters, transcription start sites (+1) and Rho-independent terminators were predicted for *S. meliloti 1021 *pSymA and pSymB (Table 4) and confirmed from conserved positions in the alignment. Click here for file
