# Supplementary figures and images for: Prediction of Sinorhizobium meliloti sRNA genes and experimental detection in strain 2011
Source: BMC Genomics. 2008 Sep 16;9:416. doi: 10.1186/1471-2164-9-416 (PMC2573895; doi:10.1186/1471-2164-9-416)

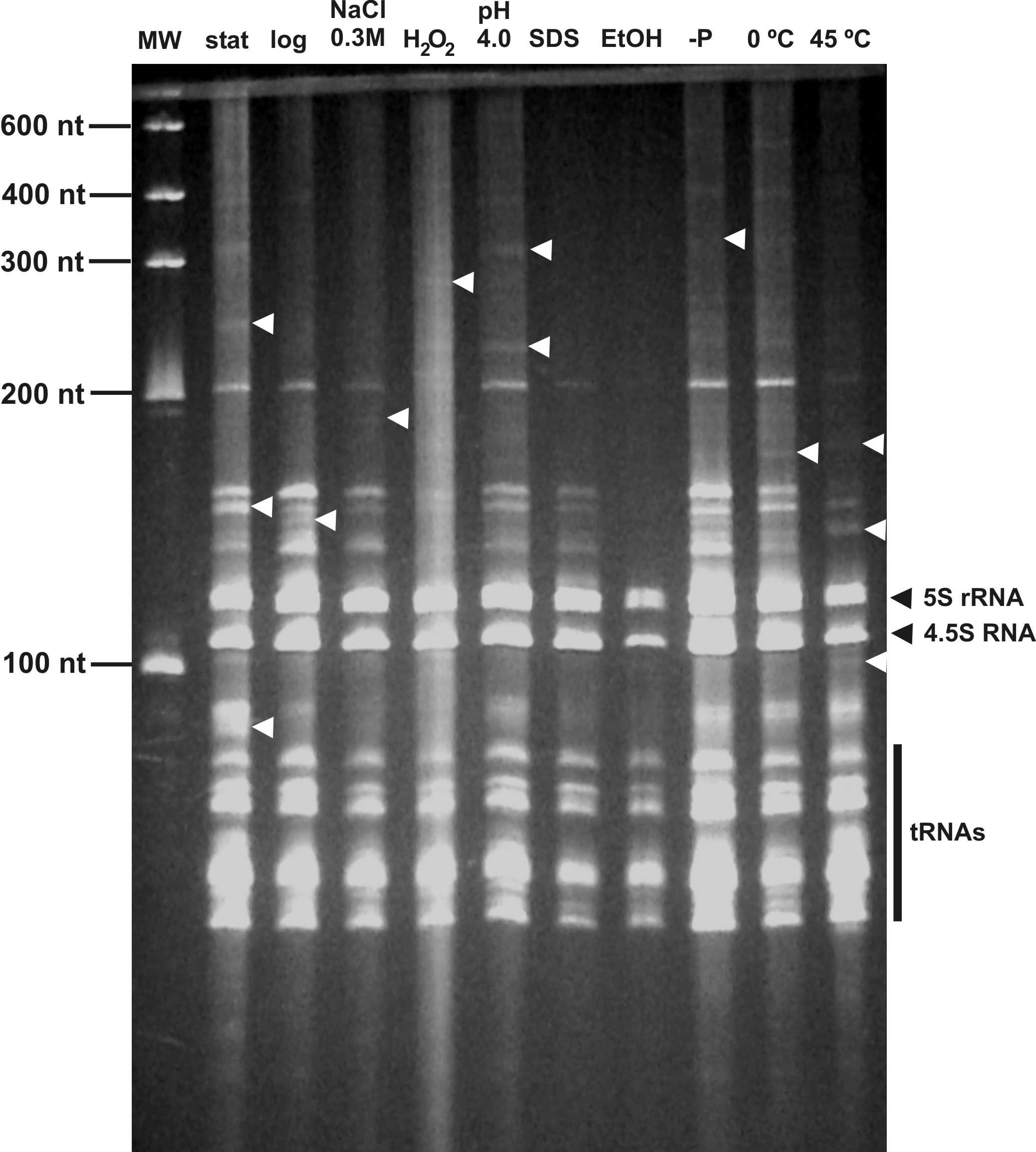

Supplement: Additional file 3 — Denaturing PAGE fractionation of S. meliloti 2011 total RNA. Electrophoretic pattern of S. meliloti 2011 total RNA in a denaturing polyacrylamide gel (8.3 M urea, 8% acrylamide and 0.2% bisacrylamide in 1× TBE buffer; 25 cm-long). Approximately 20–60 μg of total RNA, corresponding to all cells present in 20 ml of RDM cultures, were loaded in each lane. The gel was stained with ethidium bromide and visualized on an UV transilluminator. Under this conditions, effective fractionation of RNAs < 600 nt was achieved. RNA bands of varying intensity in different samples are indicated with arrowheads. Stat, stationary phase cells; log, exponential phase cells; NaCl 0.3 M, saline stress, H2O2, oxidative stress; pH 4.0, acid stress; SDS and EtOH, membrane stress; -P, phosphate starvation; 0°C, cold shock; 45°C, heat shock. [file 1471-2164-9-416-S3.jpeg]

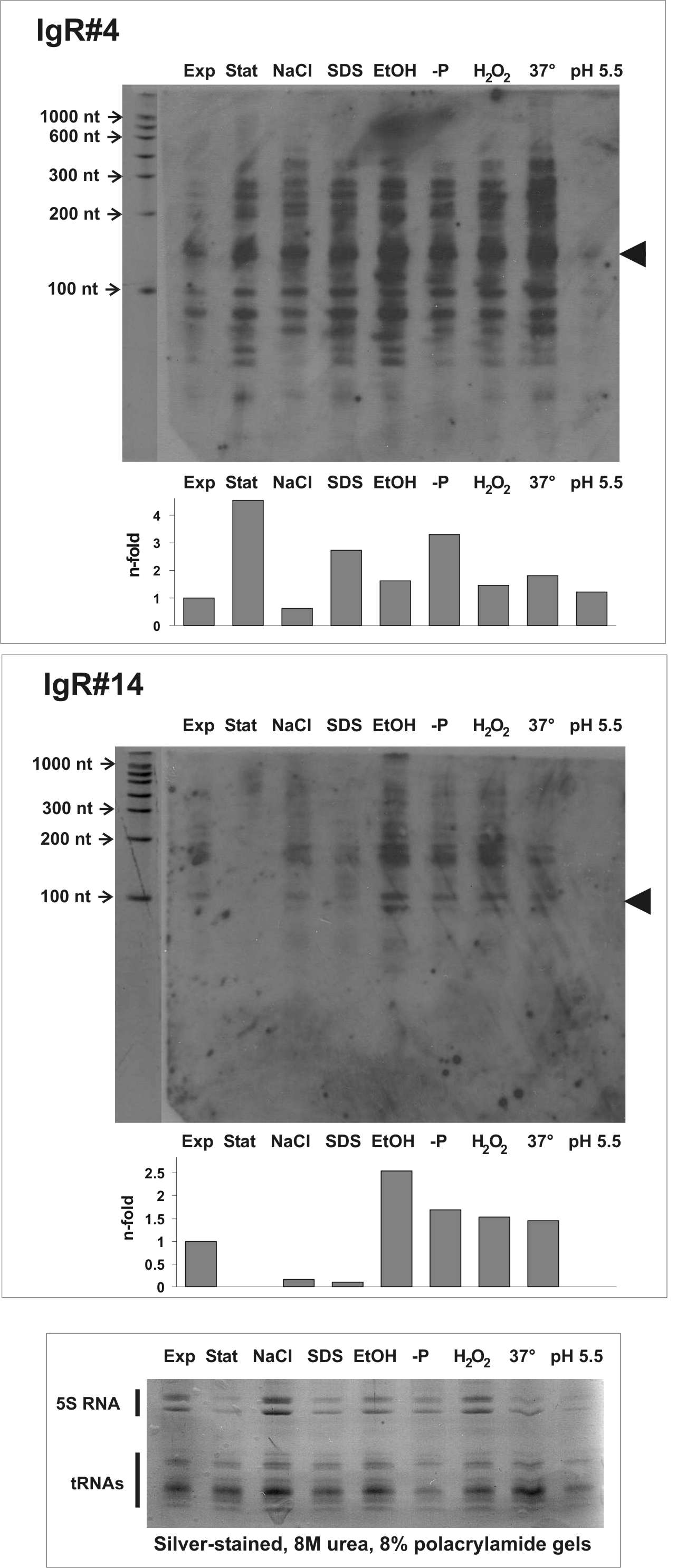

Supplement: Additional file 5 — Expression of putative sRNAs in IgR#4 and IgR#14. Northern blot analysis of putative sRNAs encoded in IgR#4 and IgR#14. See legend to Figure 1 for details. [file 1471-2164-9-416-S5.jpeg]

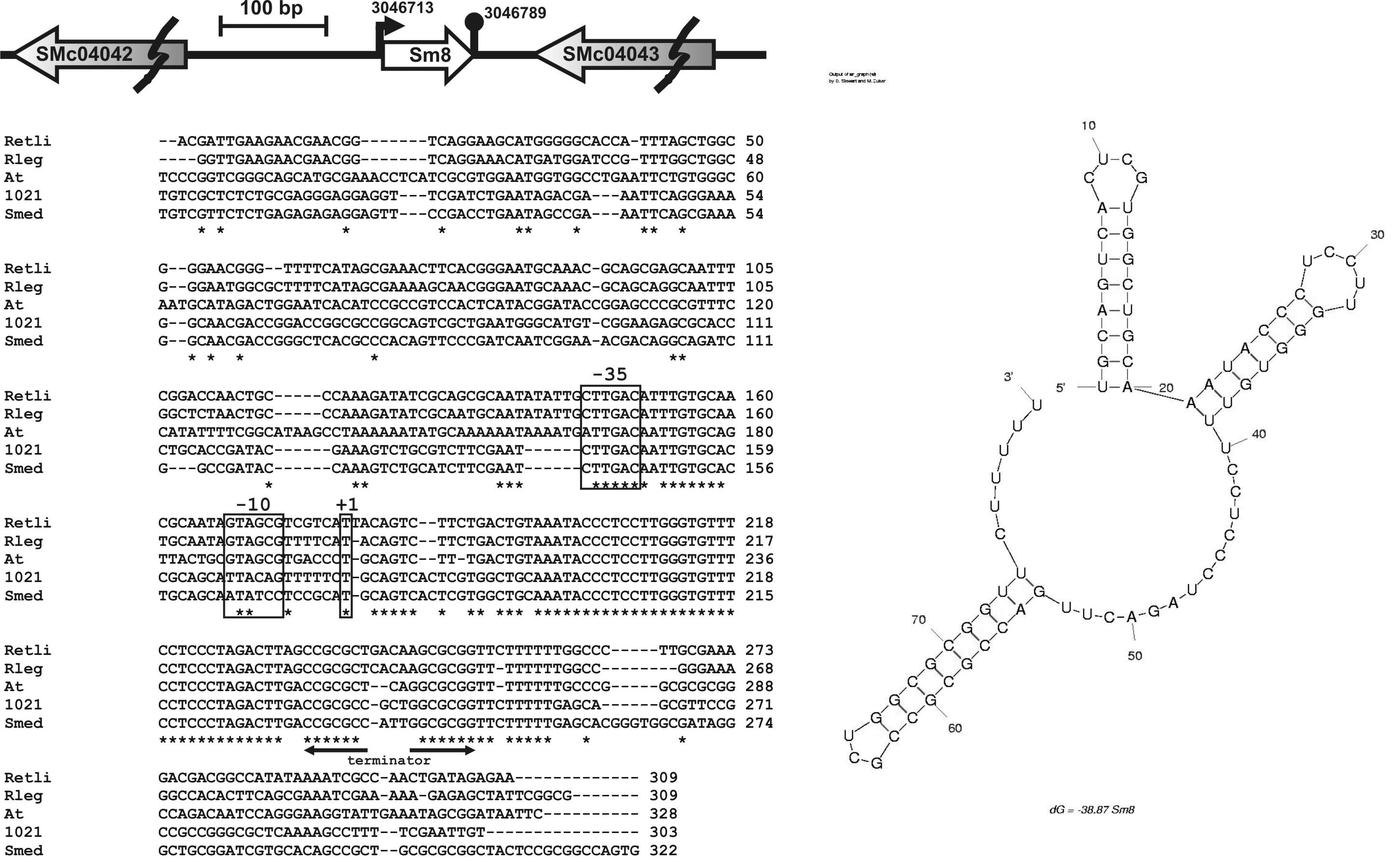

Supplement: Additional file 6 — Novel candidate sRNA gene sm8 in IgR#2. Conservation of the novel candidate sRNA gene sm8 (IgR#2) in α-proteobacteria. Sequence alignment generated with ClustalW for the corresponding IgRs of S. meliloti 1021 (1021), Sinorhizobium medicae WSM419 (Smed), Agrobacterium tumenfaciens C58 (At), Rhizobium etli CFN42 (Retli) and Rhizobium leguminosarum bv viciae 3841 (Rleg). The Rho-independent terminator was predicted for S. meliloti 1021 (see text) and confirmed from conserved positions in the alignment. The putative sigma 70-dependent promoter (-10 and -35 hexamers) and transcription start site (+1) were deduced from conserved positions in the alignment. The secondary structure presented for S. meliloti Sm8 RNA was calculated with the Mfold server [75] and corresponds to the predicted structure with lower free energy. [file 1471-2164-9-416-S6.jpeg]

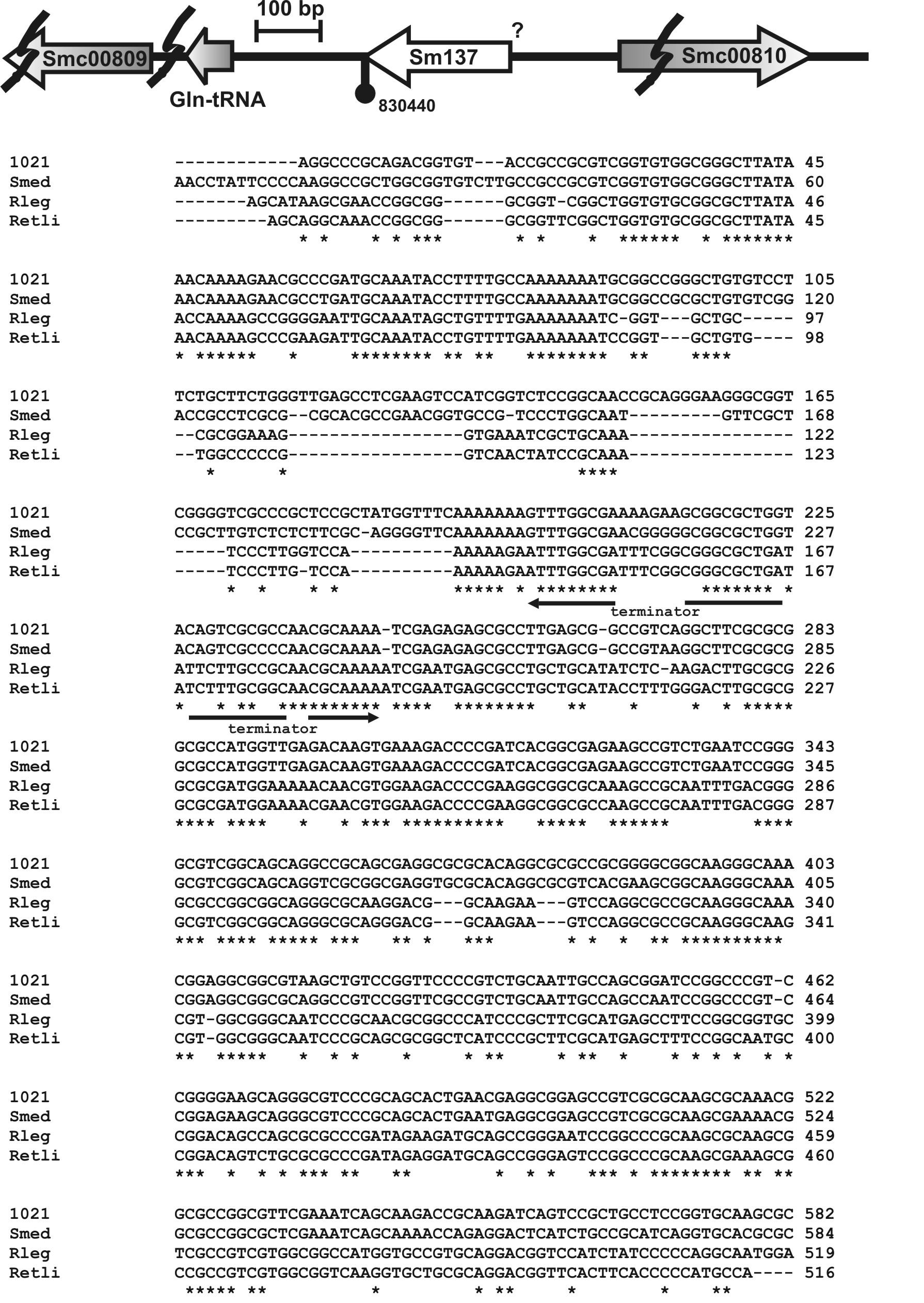

Supplement: Additional file 7 — Novel candidate sRNA gene sm137 in IgR#4. Conservation of the novel candidate sRNA gene sm137 (IgR#4) in α-proteobacteria. Sequence alignment generated with ClustalW for the corresponding IgRs of S. meliloti 1021 (1021), S. medicae WSM419 (Smed), R. etli CFN42 (Retli) and R. leguminosarum bv viciae 3841 (Rleg). The Rho-independent terminator was predicted for S. meliloti 1021 (see text) and confirmed from conserved positions in the alignment, but there was no prediction of a promoter in this IgR. [file 1471-2164-9-416-S7.jpeg]

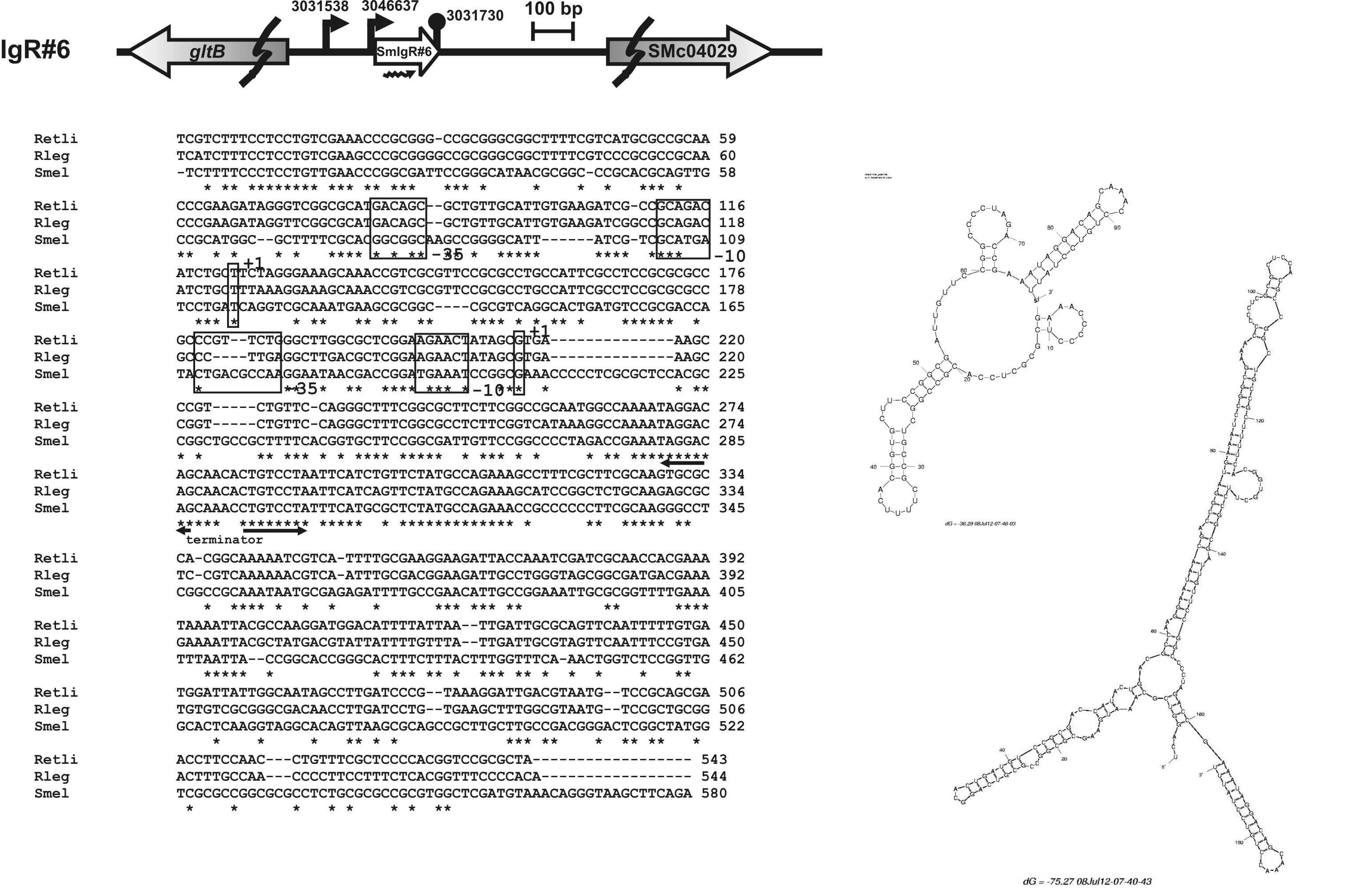

Supplement: Additional file 8 — Novel candidate sRNA gene smIgR#6. Conservation of the novel candidate sRNA gene smIgR#6 in α-proteobacteria. Sequence alignment generated with ClustalW for the corresponding IgRs of S. meliloti 1021 (1021), R. etli CFN42 (Retli) and R. leguminosarum bv viciae 3841 (Rleg). Two putative sigma 70-dependent promoters (-10 and -35 hexamers), transcription start sites (+1) and a single Rho-independent terminator were predicted for S. meliloti 1021 (see text). The secondary structure presented for both possible S. meliloti sRNAs from IgR#6 were calculated with the Mfold server [75] and correspond to the predicted structures with lower free energy. [file 1471-2164-9-416-S8.jpeg]

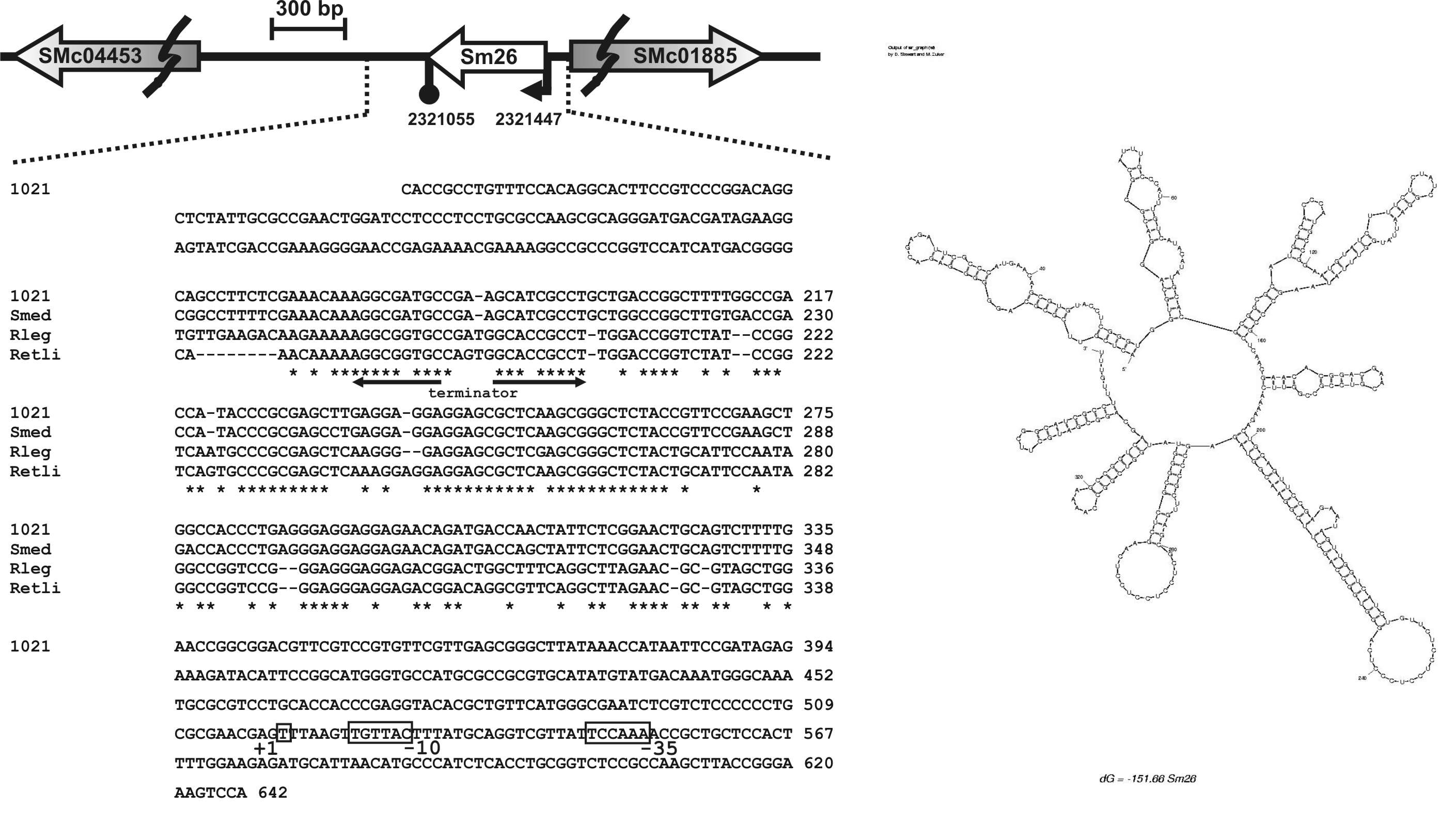

Supplement: Additional file 9 — Novel candidate sRNA gene sm26 in IgR#7. Conservation of the novel candidate sRNA gene sm26 (IgR#7) in α-proteobacteria. Sequence alignment generated with ClustalW for the corresponding IgRs of S. meliloti 1021 (1021), S. medicae WSM419 (Smed), R. etli CFN42 (Retli) and R. leguminosarum bv viciae 3841 (Rleg). The putative sigma 70-dependent promoter (-10 and -35 hexamers), transcription start site (+1) and Rho-independent terminator were predicted for S. meliloti 1021 (see text). The secondary structure presented for S. meliloti Sm26 RNA was calculated with the Mfold server [75] and corresponds to the predicted structure with lower free energy. [file 1471-2164-9-416-S9.jpeg]

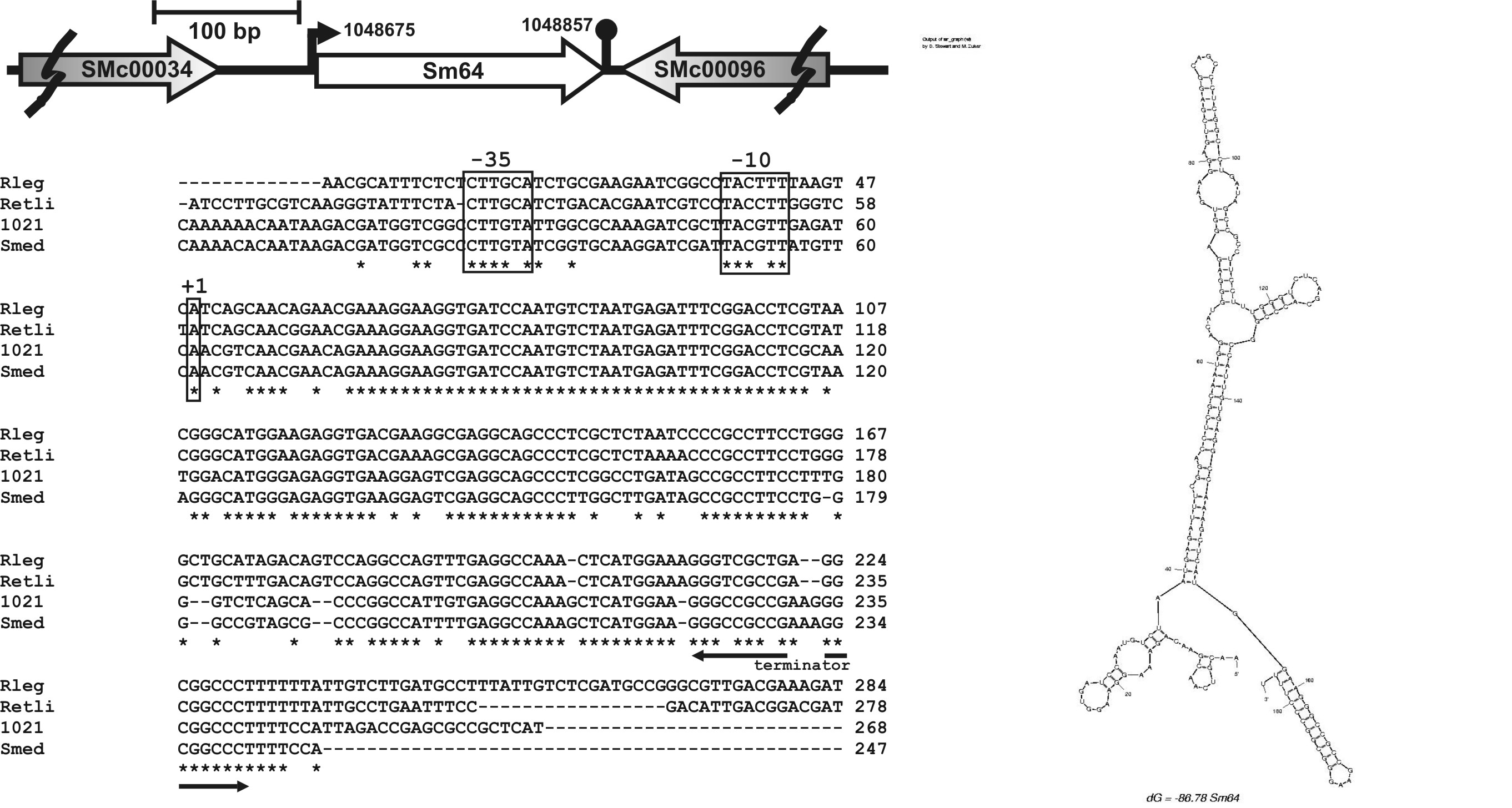

Supplement: Additional file 10 — Candidate sRNA gene sm64 (sra25) in IgR#10. Conservation of the candidate sRNA gene sm64 (IgR#10; sra25, [27]) in α-proteobacteria. Sequence alignment generated with ClustalW for the corresponding IgRs of S. meliloti 1021 (1021), S. medicae WSM419 (Smed), R. etli CFN42 (Retli) and R. leguminosarum bv viciae 3841 (Rleg). The putative sigma 70-dependent promoter (-10 and -35 hexamers), transcription start site (+1) and Rho-independent terminator were predicted for S. meliloti 1021 (see text) and confirmed from conserved positions in the alignment. The secondary structure presented for S. meliloti Sm64 RNA was calculated with the Mfold server [75] and corresponds to the predicted structure with lower free energy. [file 1471-2164-9-416-S10.jpeg]

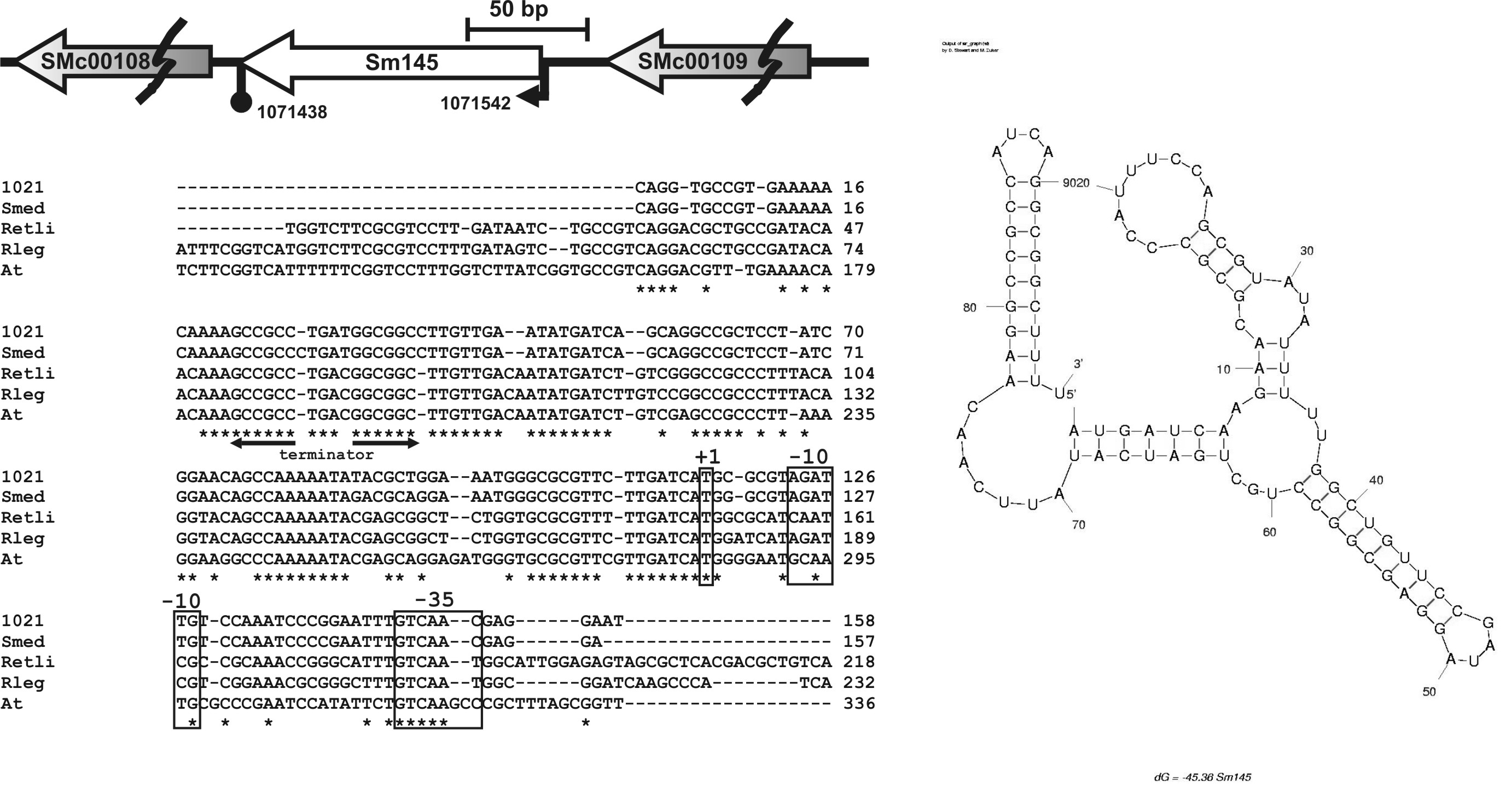

Supplement: Additional file 11 — Novel candidate sRNA gene sm145 in IgR#11. Conservation of the novel candidate sRNA gene sm145 (IgR#11) in α-proteobacteria. Sequence alignment generated with ClustalW for the corresponding IgRs of S. meliloti 1021 (1021), S. medicae WSM419 (Smed), A. tumenfaciens C58 (At), R. etli CFN42 (Retli) and R. leguminosarum bv viciae 3841 (Rleg). The putative sigma 70-dependent promoter (-10 and -35 hexamers), transcription start site (+1) and Rho-independent terminator were predicted for S. meliloti 1021 (see text) and confirmed from conserved positions in the alignment. The secondary structure presented for S. meliloti Sm145 RNA was calculated with the Mfold server [75] and corresponds to the predicted structure with lower free energy. [file 1471-2164-9-416-S11.jpeg]

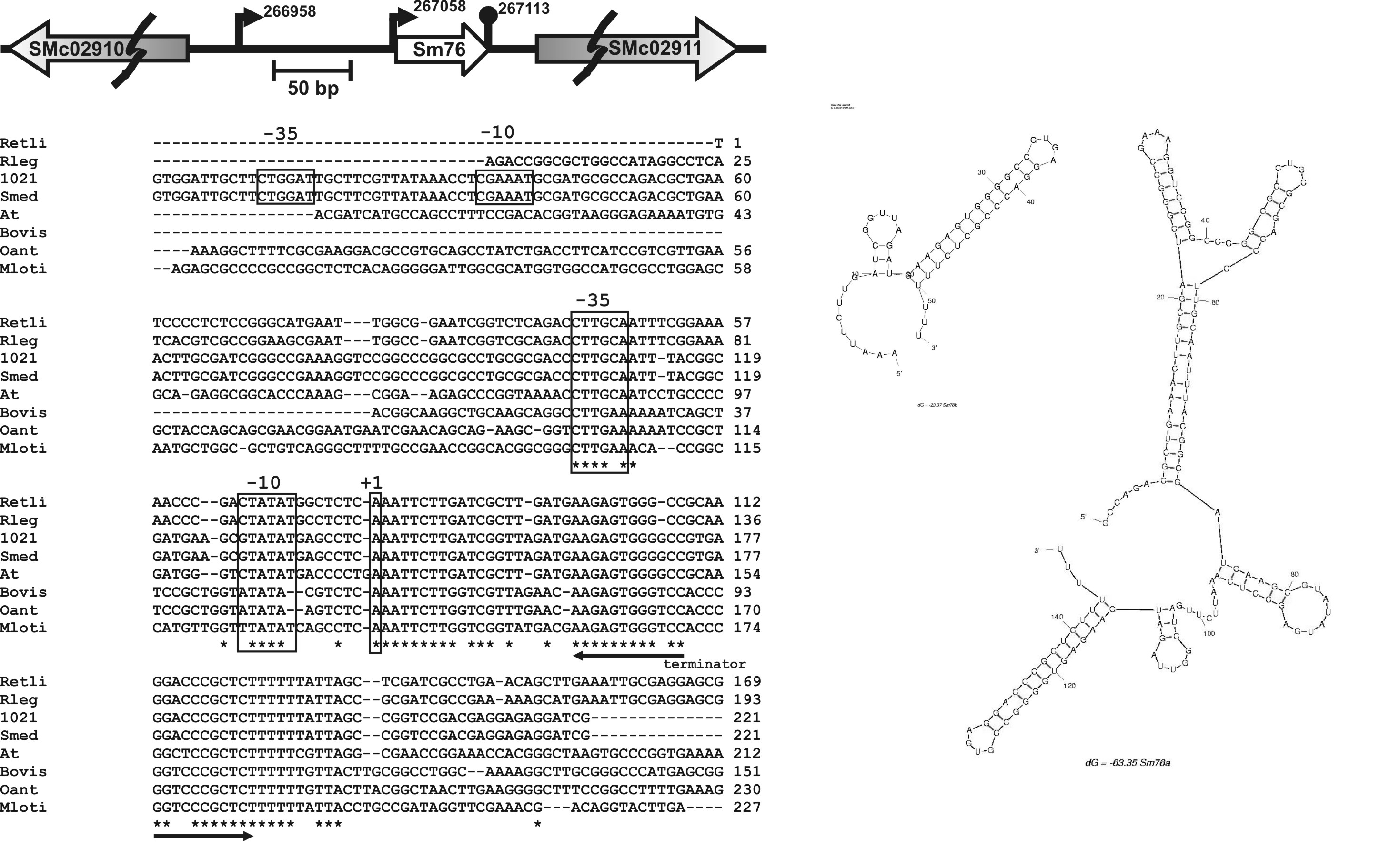

Supplement: Additional file 12 — Novel candidate sRNA gene sm76 in IgR#14. Conservation of the novel candidate sRNA gene sm76 (IgR#14) in α-proteobacteria. Sequence alignment generated with ClustalW for the corresponding IgRs of S. meliloti 1021 (1021), S. medicae WSM419 (Smed), A. tumenfaciens C58 (At), R. etli CFN42 (Retli), R. leguminosarum bv viciae 3841 (Rleg), Mesorhizobium loti MAFF303099 (Mloti), Ochrobactrum anthropi ATCC49188 (Oa) and Brucella ovis ATCC25840 (Bo). The putative sigma 70-dependent promoter (-10 and -35 hexamers), transcription start site (+1) and Rho-independent terminator were predicted for S. meliloti 1021 (see text) and confirmed from conserved positions in the alignment. A second putative promoter was predicted for S. meliloti upstream then conserved one, but it seems to be specific for Sinorhizobium. The alternative secondary structures presented for S. meliloti Sm76 RNA were calculated with the Mfold server [75] and corresponds to the predicted structure with lower free energy for the two possible transcripts. [file 1471-2164-9-416-S12.jpeg]

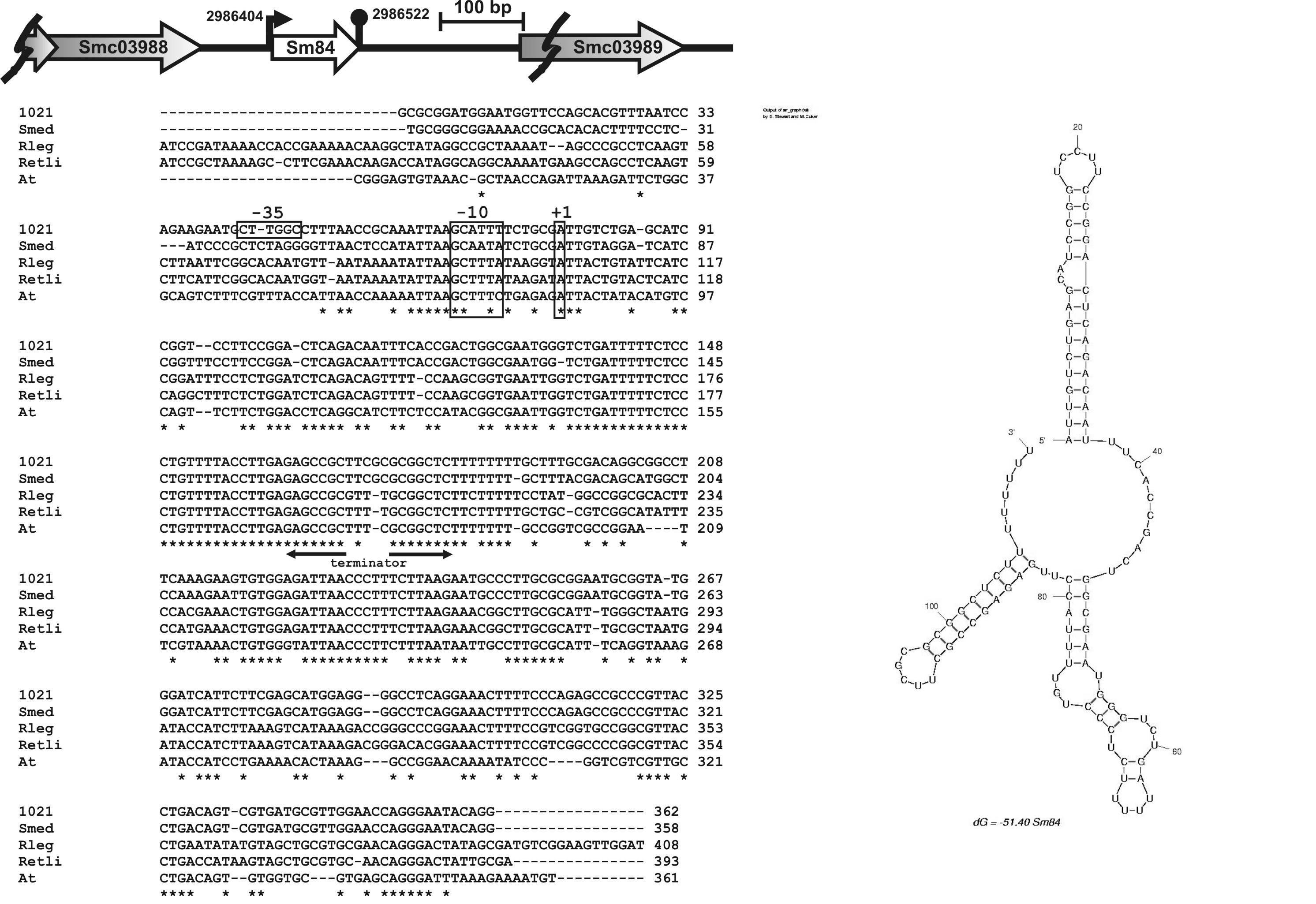

Supplement: Additional file 13 — Novel candidate sRNA gene sm84 in IgR#15. Conservation of the novel candidate sRNA gene sm84 (IgR# 15) in α-proteobacteria. Sequence alignment generated with ClustalW for the corresponding IgRs of S. meliloti 1021 (1021), S. medicae WSM419 (Smed), A. tumenfaciens C58 (At), R. etli CFN42 (Retli) and R. leguminosarum bv viciae 3841 (Rleg). The putative sigma 70-dependent promoter (-10 and -35 hexamers), transcription start site (+1) and Rho-independent terminator were predicted for S. meliloti 1021 (see text) and confirmed from conserved positions in the alignment. The secondary structure presented for S. meliloti Sm84 RNA was calculated with the Mfold server [75] and corresponds to the predicted structure with lower free energy. [file 1471-2164-9-416-S13.jpeg]

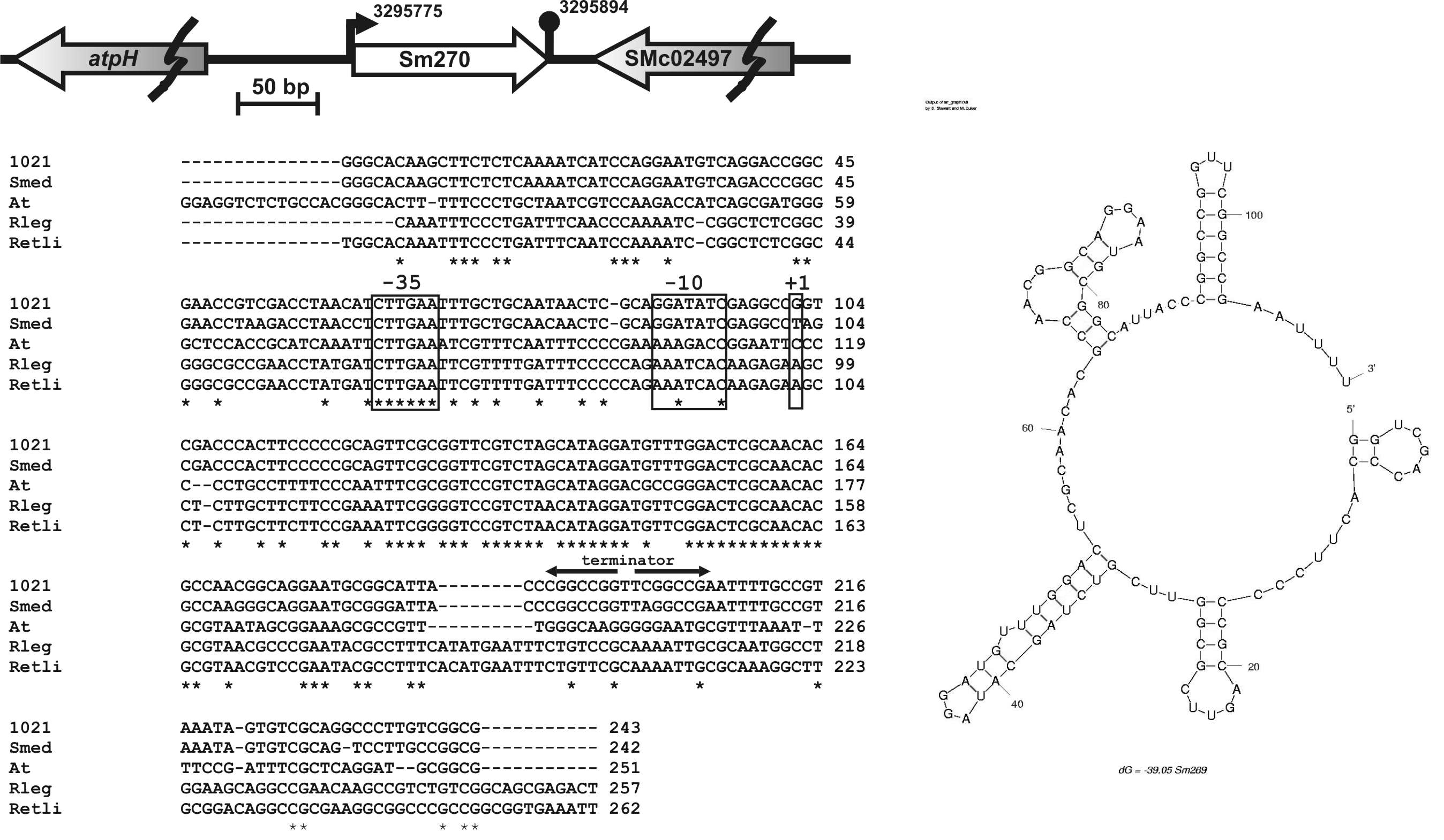

Supplement: Additional file 14 — Novel candidate sRNA gene sm270 in IgR#16. Conservation of the novel candidate sRNA gene sm270 (IgR#16) in α-proteobacteria. Sequence alignment generated with ClustalW for the corresponding IgRs of S. meliloti 1021 (1021), S. medicae WSM419 (Smed), A. tumenfaciens C58 (At), R. etli CFN42 (Retli) and R. leguminosarum bv viciae 3841 (Rleg). The putative sigma 70-dependent promoter (-10 and -35 hexamers), transcription start site (+1) and the putative Rho-independent terminator were predicted for S. meliloti 1021 (see text) and confirmed from conserved positions in the alignment. The secondary structure presented for S. meliloti Sm270 RNA was calculated with the Mfold server [75] and corresponds to the predicted structure with lower free energy. [file 1471-2164-9-416-S14.jpeg]

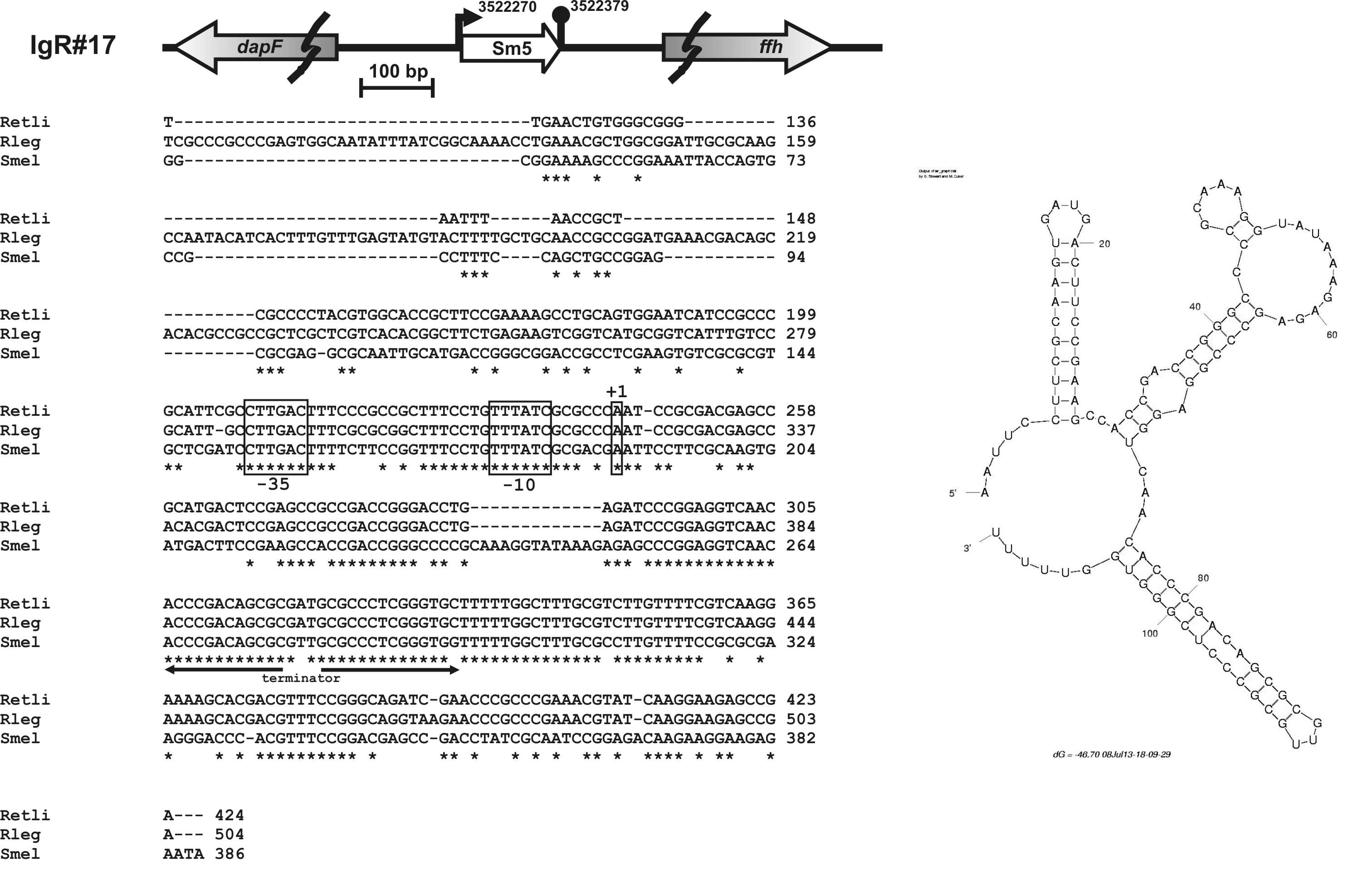

Supplement: Additional file 15 — Novel candidate sRNA gene sm5 in IgR#17. Conservation of the novel candidate sRNA gene sm5 (IgR#17) in α-proteobacteria. Sequence alignment generated with ClustalW for the corresponding IgRs of S. meliloti 1021 (1021), R. etli CFN42 (Retli) and R. leguminosarum bv viciae 3841 (Rleg). The putative sigma 70-dependent promoter (-10 and -35 hexamers), transcription start site (+1) and the putative Rho-independent terminator were predicted for S. meliloti 1021 (see text) and confirmed from conserved positions in the alignment. The secondary structure presented for S. meliloti Sm5 RNA was calculated with the Mfold server [75] and corresponds to the predicted structure with lower free energy. [file 1471-2164-9-416-S15.jpeg]

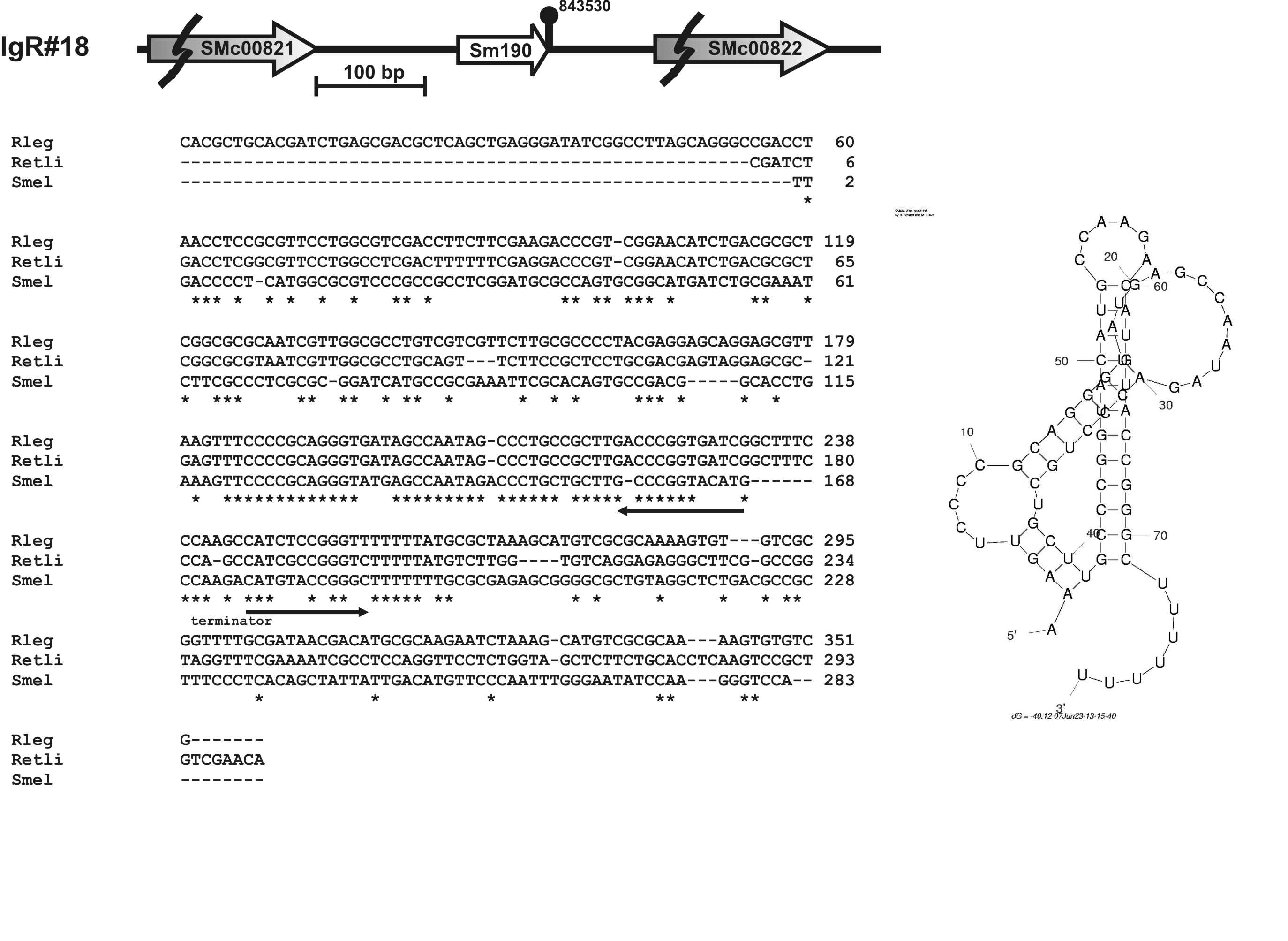

Supplement: Additional file 16 — Novel candidate sRNA gene sm190 in IgR#18. Conservation of the novel candidate sRNA gene sm190 (IgR#18) in α-proteobacteria. Sequence alignment generated with ClustalW for the corresponding IgRs of S. meliloti 1021 (1021), R. etli CFN42 (Retli) and R. leguminosarum bv viciae 3841 (Rleg). The putative Rho-independent terminator was predicted for S. meliloti 1021 (see text) and confirmed from conserved positions in the alignment. The secondary structure presented for S. meliloti Sm190 RNA was calculated with the Mfold server [75] and corresponds to the predicted structure with lower free energy assuming that the sRNA extends along the conserved sequence upstream the terminator and includes the terminator itself. [file 1471-2164-9-416-S16.jpeg]

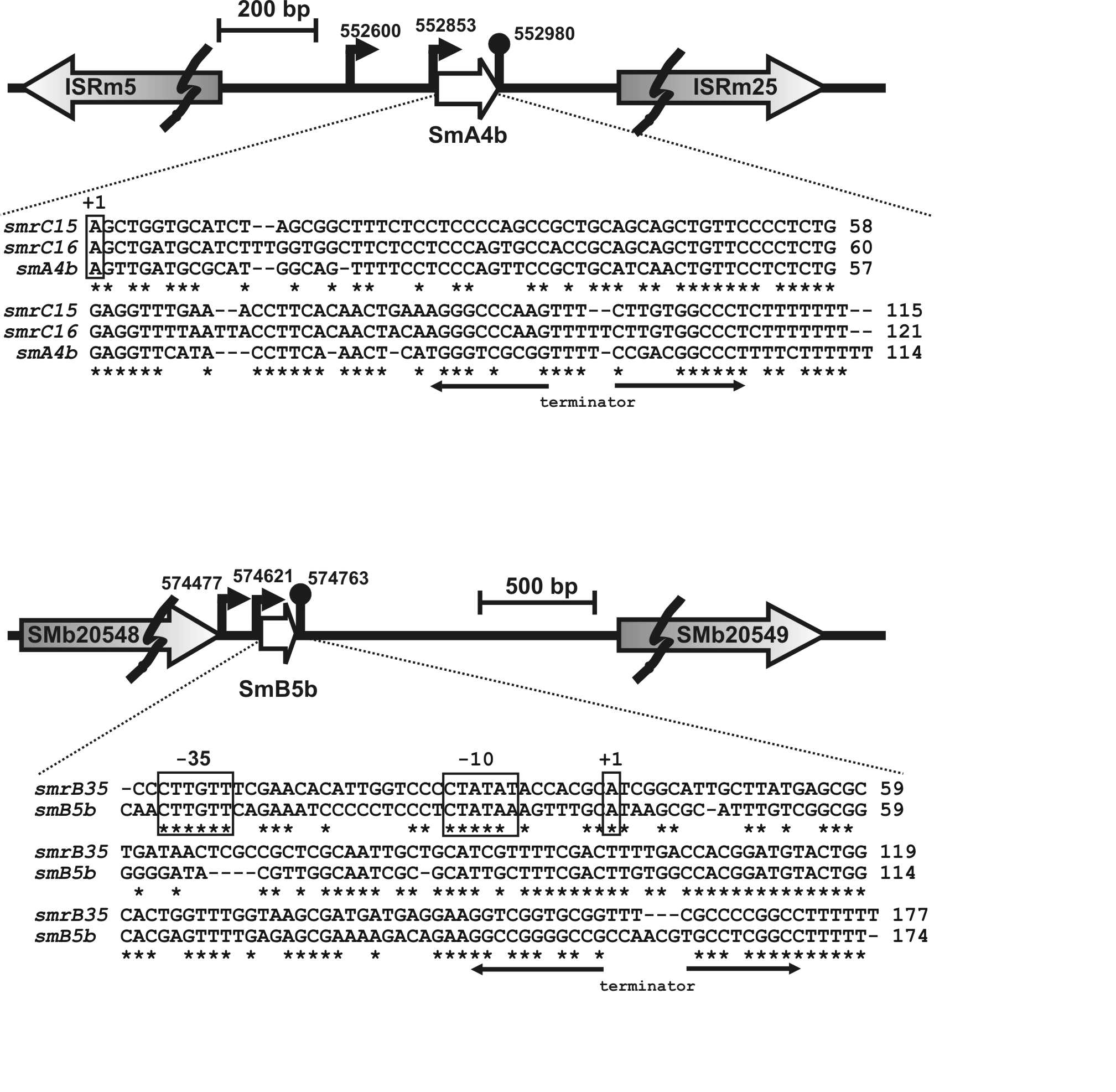

Supplement: Additional file 19 — Homologs of sRNA genes smrC14-smrC15 and smrB35 are present in pSymA and pSymB IgRs. Identification of extra copies of sRNA genes smrC14-smrC15 and smrB35 in pSymA and pSymB IgRs. Genetic surroundings and sequence alignments generated with ClustalW for the sRNA genes of S. meliloti 1021 smrC14, smrC15 [26] and the corresponding identified homolog in pSymA (smA4b; Table 4), and for the sRNA gene smrB35 [26] and the corresponding identified homolog in pSymB (smB5b; Table 4). The putative sigma 70-dependent promoters, transcription start sites (+1) and Rho-independent terminators were predicted for S. meliloti 1021 pSymA and pSymB (Table 4) and confirmed from conserved positions in the alignment. [file 1471-2164-9-416-S19.jpeg]
