# Supplementary material for: Prediction of Sinorhizobium meliloti sRNA genes and experimental detection in strain 2011
Source: BMC Genomics. 2008 Sep 16;9:416. doi: 10.1186/1471-2164-9-416 (PMC2573895; doi:10.1186/1471-2164-9-416)
Supplement: Additional file 18 — Predicted transcriptional units in S. meliloti 1021 chromosomal IgRs. sRNA candidates identified as putative transcriptional units in intergenic regions of S. meliloti 1021 chromosome. [file 1471-2164-9-416-S18.doc]

**Table S4.** sRNA candidates identified as putative transcriptional units in intergenic regions of *S. meliloti* 1021 chromosome according to the global scoring procedure.

| **sRNA# ^1^** | **Gene or**  **designation ^2^** | **IGR length** | **IGR start** | **Upstream ORF** | **Orientation**  **Up sRNA Dn** | | | **Promoter prediction ^3^** | **Predicted 5'-end ^4^** | **Predicted 3'-end ^5^** | **Blast score** | **Qrna score** | **Global score** | **Length (nt) ^5^** | **SP ^7^** |
| --- | --- | --- | --- | --- | --- | --- | --- | --- | --- | --- | --- | --- | --- | --- | --- |
| 1 | **SmrC15 (Sm3) SmrC16 (Sm3’)** | 652 | 1698492 | SMc01226 | < < | <  < | < < | NN, s70  PhoB | 1698732  1698968 * | 1698618  1698820 | 6 | 2 | 98 | 114  151 | y |
| 2 | Sm4 | 541 | 2371344 | SMc01844 | > | > | < | NN, s70 | 2371490, 2371606 | 2371852 | 1 | 1 | 82 | 256-362 | n |
| **3** | Sm5 (*ffh* leader?) | 384 | 3522082 | SMc03856 | < | > | > | s70 | 3522121, 3522271 | 3522379 | 12 | 8 | 80 | 108-258 | y |
| 4 | Sm6 | 491 | 1727920 | SMc01202 | < | < | < | NN, s70 | 1728153, 1728196, 1728269 | 1728021 | 1 | 1 | 62 | 120-248 | n |
| 5 | **SmrC14 (Sm7)**  Sm7’ | 922 | 1667435 | SMc02051 | < < | <  < | < < | NN, s70  NN | 1667614  1667983 | 1667488  1667769 | 3 | 5 | 58 | 126  214 | n |
| **6** | **Sm8** | 301 | 3046532 | SMc04042 | < | > | < | NN, s70 | 3046713 | 3046789 | 9 | 4 | 53 | 76 | y |
| 7 | Sm9 | 470 | 1635180 | SMc02080 | > | < | > | NN, s70 | 1635343, 1635411, 1635564 | 1635217 | 1 | 1 | 52 | 126-347 | n |
| 8 | Sm10 | 405 | 1090966 | SMc00057 | > | > | > | NN, s70 | 1091047, 1091106 | 1091343 | 1 | 1 | 52 | 237-296 | n |
| **9** | **SmrC9 (Sm12)** | 710 | 1398105 | SMc01933 | < | < | > | NN, s70 | 1398427, 1398584 | 1398279 | 4 | 3 | 47 | 148-305 | y |
| **10** | **SmrC7 (Sm13)** | 472 | 201484 | SMc02850 | < | > | > | NN, s70 | 201682 | 201829 | 4 | 3 | 47 | 147 | y |
| 11 | Sm11 | 1101 | 2474890 | SMc01671 | < | > | < | NN, s70 | 2475717, 2475862 | 2475961 | 5 | 2 | 47 | 99-244 | y |
| 12 | Sm14 | 410 | 573683 | SMc02139 | < | > | > | NN, PhoB | 573816, 573830 * | 574040 | 4 | 1 | 45 | 210 | y |
| **13** | **Sm17** | 829 | 560580 | SMc02151 | < | > | < | NN, s70 | 560780, 560916, 561000 | 561258 | 1 | 1 | 42 | 258-478 | n |
| 14 | Sm16 | 902 | 1197909 | SMc02597 | > | < | > | NN, s70 | 1198309, 1198440, 1198508 | 1198093 | 1 | 1 | 42 | 212-416 | n |
| 15 | Sm18 | 277 | 2270948 | SMc01425 | < | > | > | s70 | 2270996, 2271068 | 2271224 | 1 | 1 | 42 | 156-228 | n |
| 16 | Sm23 | 292 | 1706663 | SMc01218 | < | < | > | NN | 1706863 | 1706715 | 1 | 3 | 34 | 148 | n |
| 17 | Sm25 | 396 | 2210147 | SMc04289 | < | > | > | s70, PhoB | 2210185, 2210260 * | 2210322 | 1 | 2 | 33 | 62-137 | n |
| **18** | **Sm26** | 1270 | 2320296 | SMc04453 | < | < | > | NN | 2321447 | 2321055 | 2 | 1 | 33 | 392 | y |
| 19 | Sm28 | 826 | 713263 | SMc03014 | > | < | > | NN, s70 | 713461, 713680, 713763 | 713290 | 1 | 1 | 32 | 170-463 | n |
| **20** | Sm30 | 644 | 1518568 | SMc01257 | < | > | > | NN, s70, PhoB | 1518613, 1518748 * | 1518988 | 1 | 1 | 32 | 234-375 | n |
| 21 | Sm29 | 279 | 2059615 | SMc04232 | > | < | < | NN | 2059821 | 2059696 | 1 | 1 | 32 | 125 | n |
| 22 | Sm31 (*rpsK* leader?) | 226 | 1491104 | SMc01287 | > | < | > | NN | 1491231 | 1491112 | 10 | 1 | 31 | 119 | n |
| 23 | Sm39 | 351 | 523541 | SMc02172 | < | > | > | NN, s70 | 523806 | 523874 | 3 | 1 | 24 | 68 | y |
| 24 | Sm38 | 609 | 2594925 | SMc04457 | > | > | > | NN | 2595346 | 2595464 | 3 | 1 | 24 | 118 | n |
| 25 | Sm43 | 488 | 1131947 | SMc02377 | > | < | > | NN, s70 | 1132163 | 1132080 | 1 | 2 | 23 | 83 | n |
| 26 | Sm44 | 299 | 2489886 | SMc01749 | < | < | > | s54 | 2490145 * | 2490042 | 2 | 1 | 23 | 93 | n |
| 27 | Sm56 | 514 | 1093054 | SMc00456 | < | > | > | NN, s70 | 1093212 | 1093442 | 1 | 1 | 22 | 230 | n |
| 28 | Sm53 | 246 | 1246045 | SMc00575 | > | < | > | s70 | 1246195 | 1246099 | 1 | 1 | 22 | 96 | n |
| 29 | Sm55 | 271 | 1599197 | SMc02111 | > | < | > | NN | 1599343 | 1599220 | 1 | 1 | 22 | 123 | n |
| 30 | Sm50 | 370 | 1636940 | SMc02079 | > | < | > | s70 | 1637112 1637180 | 1636963 | 1 | 1 | 22 | 149-217 | y |
| 31 | Sm45 | 807 | 1734329 | SMc01196 | > | > | < | NN, PhoB | 1734398, 1734337 * | 1734632 | 1 | 1 | 22 | 234 | n |
| 32 | Sm48 | 905 | 1822801 | SMc00253 | > | > | > | s70 | 1823011 | 1823233 | 1 | 1 | 22 | 222 | n |
| 33 | Sm57 | 261 | 1825826 | SMc00256 | < | > | > | NN | 1825896 | 1825995 | 1 | 1 | 22 | 99 | n |
| 34 | Sm51 | 350 | 1924386 | SMc00538 | > | < | > | NN | 1924616 | 1924418 | 1 | 1 | 22 | 198 | n |
| 35 | Sm52 | 313 | 2436480 | SMc01641 | > | < | > | NN | 2436602, 2436690 | 2436480 | 1 | 1 | 22 | 122-210 | n |
| 36 | Sm54 | 265 | 2947352 | SMc02942 | < | > | < | NN | 2947432 | 2947576 | 1 | 1 | 22 | 144 | n |
| **37** | **Sm76** | 222 | 266919 | SMc02910 | < | > | > | s70 | 266958 | 267113 | 3 | 4 | 17 | 155 | y |
| **38** | **Sm84** | 363 | 2986333 | SMc03988 | > | > | > | s70 | 2986404 | 2986522 | 4 | 2 | 16 | 118 | y |
| 39 | Sm104 | 273 | 2575787 | SMc02725 | < | < | < | s70 | 2575912 | 2575835 | 2 | 2 | 14 | 77 | y |
| 40 | Sm103 | 305 | 3129922 | SMc03168 | < | < | > | s70 | 3130151 | 3131065 | 1 | 3 | 14 | 86 | n |
| 41 | Sm105 | 264 | 658596 | SMc02285 | > | < | > | NN | 658768 | 658639 | 2 | 1 | 13 | 129 | n |
| 42 | Sm134 | 203 | 363981 | SMc00414 | < | > | < | NN | 364074 | 364166 | 1 | 1 | 12 | 92 | n |
| 43 | Sm131 | 278 | 424089 | SMc01127 | > | > | < | NN | 424155 | 424271 | 1 | 1 | 12 | 116 | n |
| 44 | Sm139 | 317 | 757439 | SMc03060 | < | > | > | s70 | 757523 | 757741 | 1 | 1 | 12 | 218 | n |
| **45** | **Sm145** | 159 | 1071425 | SMc00108 | < | < | < | s70 | 1071542 | 1071438 | 1 | 1 | 12 | 104 | y |
| 46 | Sm132 | 352 | 1714746 | SMc01213 | > | < | > | NN | 1714988 | 1714747 | 1 | 1 | 12 | 241 | n |
| 47 | Sm138 | 728 | 1718586 | SMc01210 | < | > | > | s70 | 1718903 | 1718810 | 1 | 1 | 12 | 93 | n |
| 48 | Sm133 | 726 | 1743796 | SMc01187 | < | < | > | NN | 1744253 | 1744106 | 1 | 1 | 12 | 147 | n |
| 49 | Sm118 | 785 | 1821077 | SMc00251 | > | > | < | s70 | 1821110 | 1821367 | 1 | 1 | 12 | 257 | n |
| 50 | Sm135 | 463 | 2129117 | SMc04310 | > | < | > | NN | 2129308 | 2129186 | 1 | 1 | 12 | 122 | n |
| 51 | Sm140 | 303 | 2305173 | SMc01458 | < | < | > | s70 | 2305251 | 2305183 | 1 | 1 | 12 | 68 | n |
| 52 | Sm136 | 192 | 2524196 | SMc01591 | > | < | > | s70 | 2524331 | 2524224 | 1 | 1 | 12 | 107 | n |
| 53 | Sm49 | 486 | 2695309 | SMc01971 | < | < | < | NN | 2695673 | 2695418 | 1 | 1 | 12 | 255 | n |
| 54 | Sm142 | 188 | 2785614 | SMc02414 | < | < | > | NN | 2785719 | 2785629 | 1 | 1 | 12 | 90 | n |
| 55 | Sm143 | 168 | 2961293 | SMc03967 | < | < | > | NN | 2961406 | 2961346 | 1 | 1 | 12 | 60 | n |
| 56 | Sm117 | 345 | 3238508 | SMc03104 | < | > | < | NN | 3238685 | 3238834 | 1 | 1 | 12 | 149 | n |
| **57** | Sm130 | 540 | 3650622 | SMc02797 | < | > | < | s70 | 3650963 | 3651141 | 1 | 1 | 12 | 178 | n |
| 58 | Sm63 | 554 | 3301080 | SMc02494 | < | < | > | NN | 3301198 | 3301118 | 1 | 0 | 11 | 80 | n |

**^1^** Candidate sRNAs are sorted according to its descending global scores (GS). Bolded candidates correspond to IGR regions selected in the first round of predictions (Table 1).

**^2^** The designation Sm1-Sm270 corresponds to the full list of sRNA predictions (Table S3) obtained in this work by weighing the prediction of transcriptional units (co-orientated promoter and terminator), transcriptional signals (promoter or terminator), conservation of primary and secondary structure. Bolded candidates have been verified experimentally and retain their original annotation.

**^3^** NN, neural network promoter prediction; s70, σ^70^-dependent promoter; s54, RpoN (σ^54^)-dependent promoter promoter; PhoB, putative PhoB binding site.

**^4^** The coordinates of all putative promoters and/or transcription factor binding sites within an IGR are presented. The position given for NN is the 3’ end of the identified sequence. For σ^70^ the position given is 7 bases downstream of the 3’ end of the -10 hexamer. The 3’ end of the predicted binding site for σ^54^ and PhoB is indicated with an asterisk.

**^5^** Given is the position of the last uridine at the end of the terminator sequence.

**^6^** Range of possible lengths based on putative 5’ and 3’ ends.

**^7^** y, candidate present in sRNAPredictHT search; n, candidate absent in sRNAPredictHT output (see Additional Table S2).
